# Supplementary material for: Wounding triggers MIRO-1 dependent mitochondrial fragmentation that accelerates epidermal wound closure through oxidative signaling
Source: Nat Commun. 2020 Feb 26;11:1050. doi: 10.1038/s41467-020-14885-x (PMC7044169; doi:10.1038/s41467-020-14885-x)
Supplement: Supplementary file 1 — Supplementary Information [file 41467_2020_14885_MOESM1_ESM.pdf]

Wounding triggers MIRO-1 dependent mitochondrial fragmentation that accelerates epidermal wound closure through oxidative signaling

Hongying Fu, Hengda Zhou, Xinghai Yu, Jingxiu Xu, Jinghua Zhou, Xinan Meng, Jianzhi Zhao, Yu Zhou, Andrew D. Chisholm, and Suhong Xu

Supplementary information includes 7 Supplementary Figures and legends, 6 Supplementary Tables, and Supplementary References.

Supplementary Figures and legends

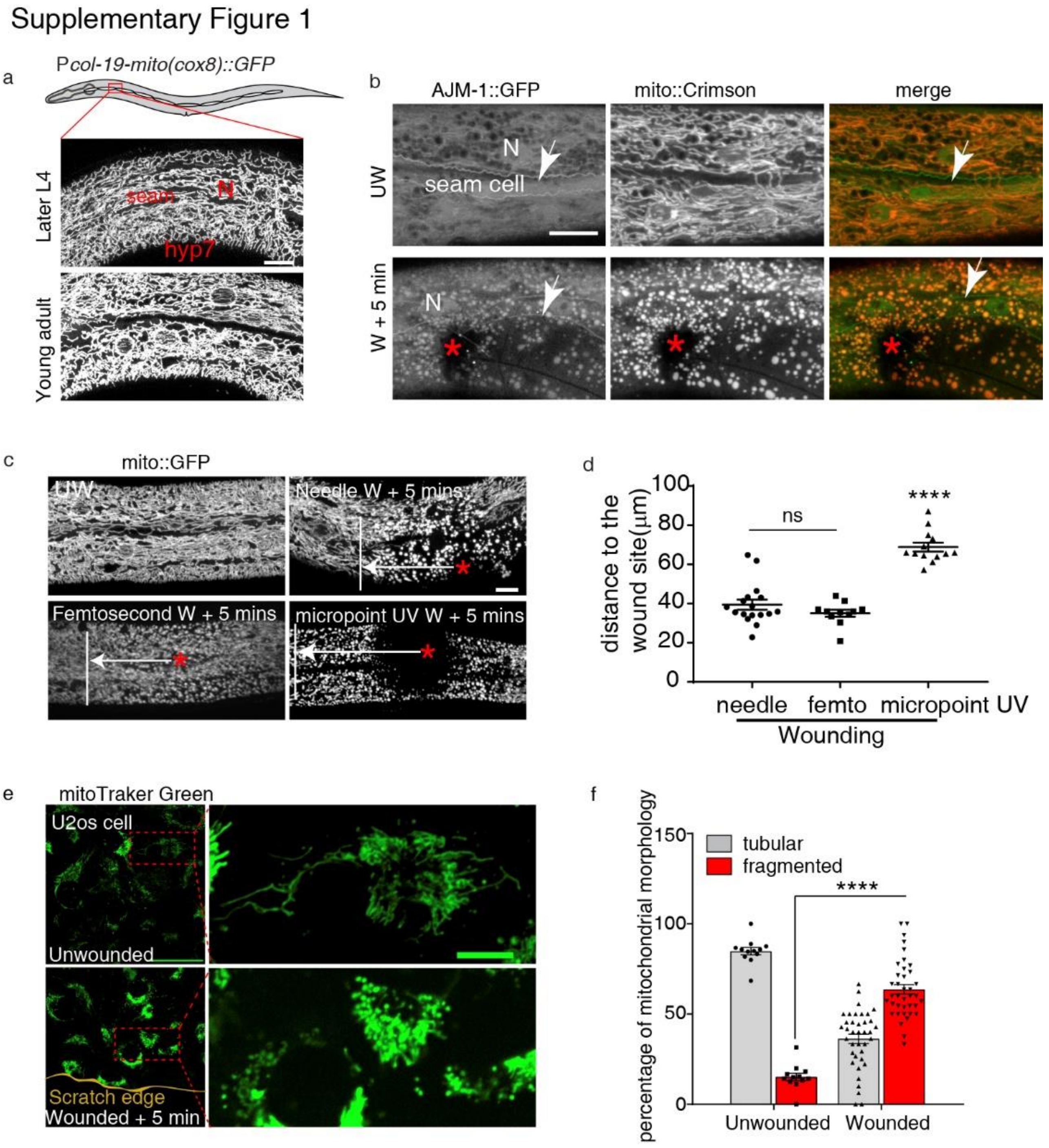

Supplementary Figure1: Wounding triggers rapid local mitochondrial fragmentation in the *C. elegans* epidermis. a, Mitochondrial network in late larval (L4) and young adult *C. elegans* epidermis. *C. elegans* epidermal mitochondria were labeled with *mito::GFP (juEx4796)* (mitochondrial target sequence of cox-8), expressed under the control of epidermal specific promoter *col-19*. We observed few mitochondrial fusion and fission events over 5 minutes in absence of wounding (See also Supplementary Movie 1). N: nucleus. **b**, Mitochondrial fragmentation at 5 min post-needle wounding in adult wild type (WT) worms. Subapical junctions were labeled with the *AJM-1::GFP(jcIs1)* transgene and mitochondria were labeled with the *mito::crimson*(*juEx4880*) transgene. N: nucleus. Red asterisks indicate

wound site, white arrow indicates *AJM-1::GFP*. **c**, Representative confocal images of epidermal mitochondria with or without wounding. Red asterisks indicate the wound sites. White arrows indicate the distance of fragmented mitochondria to the wound site. White lines indicate the farthest fragmented mitochondria, and white arrows indicate the distance of mitochondrial fragmentation. N = 5 biologically independent experiments with similar results. Scale bars (**a**-**c**): 10 μm. **d**, Quantitation of farthest distance of fragmented mitochondria to the wound site (needle, n = 17; laser, n = 11; Micropoint UV, n = 13 animals). Mitochondrial fragmentation at 5 min post-laser (femtosecond) and needle wounding in the adult epidermis of WT and can spread to around 40 μm to the wound site. Micropoint UV laser wounding triggers mitochondrial fragmentation ~70 μm to the wound site. We measured 20 lines from the wound site to the farthest fragmented mitochondria for each animal and took the average. Bars indicate mean ± SEM. ns, P = 0.4162, ****, p < 0.0001, One-way ANOVA Tukey's test. **e**, Representative confocal images of mitochondria in U2OS cell with or without scratch wounding. Mitochondria were stained with mitoTracker Green for 20 mins before the scratch wounding. Scale bars: 50 μm, enlarged images 10 μm. **f**, Quantitation of mitochondrial morphology in U2OS cells before and after scratch wounding (Unwounded, n = 12; Wounded n = 38 cells), Overall mitochondrial morphology (tubular or fragmented) in each cell was quantified by double-blind scoring. Bars indicate mean ± SEM. ****, P < 0.0001, Two-tailed unpaired *t*-test. Source data are provided as a Source Data file.

Supplementary Figure 2

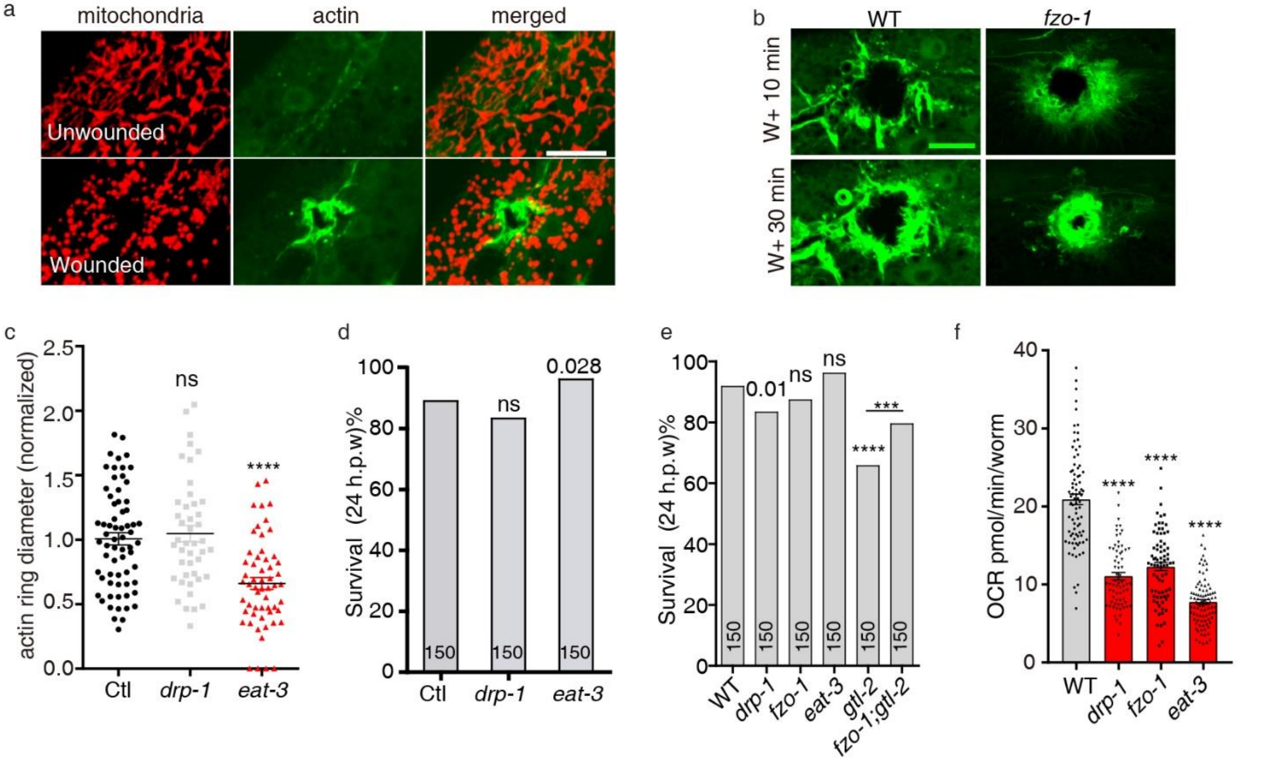

Supplementary Figure 2: Mitochondrial fragmentation mutants showed enhanced wound closure. **a**, Representative confocal images of mitochondria and actin polymerization before and after wounding. Note, actin ring is formed during the fragmented mitochondria at the wound site. See also Supplementary Movie 3. *Pcol-19-GFP::moesin(juIs352);Pcol-19-mito-mKate2(zjuSi47)* stain were used for imaging. N = 3 independent experiments. **b**, Loss of function in *fzo-1(tm1133)* showed faster wound closure (see also Supplementary Movie 4). Representative confocal images of actin ring closure 10 min and 30 min post-wounding (N = 5 independent experiment). See also Supplementary Movie 4. Scale bars (**a**,**b**): 10 μm. **c**, Knockdown *eat-3* via RNAi accelerated wound closure. Quantitation of actin ring diameter 1 h.p.w. (hour-post wounding) (Ctl, n = 66; *drp-1*, n = 44; *eat-3*, n = 57). Bars indicate mean ± SEM. ns, P = 0.8055, ****, P < 0.0001 (versus WT), One-way ANOVA Dunnett's test. **d**, Epidermal specific RNAi of *drp-1* or *eat-3* did not affect 24 h post-wounding survival; RNAi was performed using the epidermal specific RNAi strain CZ14540[*rde-1(ne219);Pcol-19-rde-1(juIs346)*]. n = 150, ns, P = 0.8055, Two-sided Fisher's exact test. **e**, Mitochondrial mutants have normal 24 h post-wounding survival and suppress *gtl-2(n2618)* low post-wounding survival. WT or mutant animals were wounded at young adult stage, live animals were counted 24 h later. The defect in survival after needle wounding of *gtl-2(n2618)* is suppressed in double mutants with *fzo-1(tm1133)*. n =150, ns, P = 0.1168 (*fzo-1*) or 0.4419 (*eat-3*), ***, P = 0.0096, ****, P < 0.0001 (vs. WT), Two-sided Fisher's exact test. **f**. Oxygen consumption rate (OCR) in WT and mitochondrial dynamics mutants of *drp-1(tm108), fzo-1(tm1133),* and *eat-3(tm1107)* (WT, n = 83; *drp-1*, n = 68; *fzo-1*, n = 77; *eat-3*, n = 88 animals). Bars indicate mean ± SEM, ****, P < 0.0001 (vs. WT), One-way ANOVA Dunnett's test. Source data are provided as a Source Data file.

Supplementary Figure 3

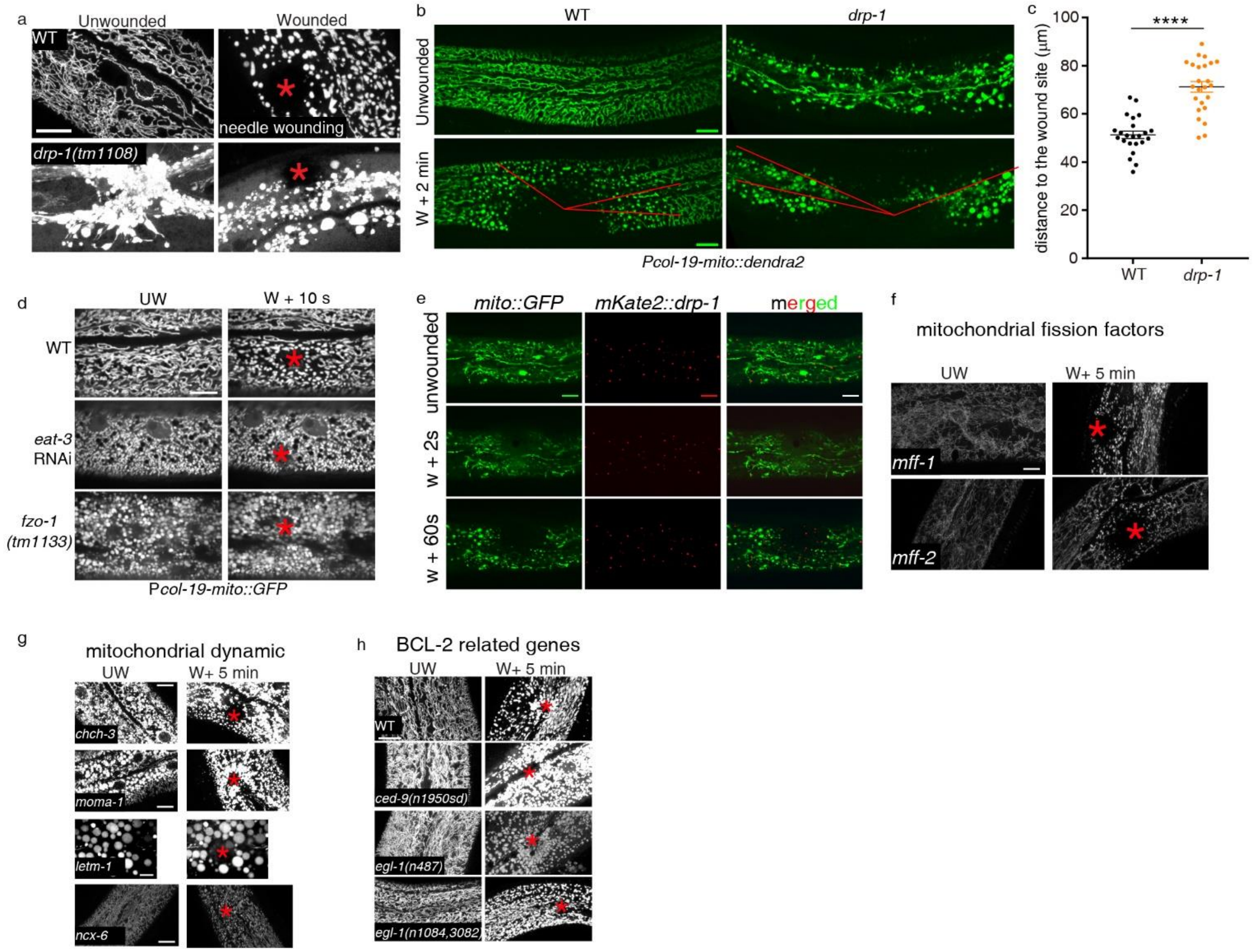

Supplementary Figure 3: Epidermal wounding triggered mitochondrial fragmentation is not dependent on canonical mitochondrial dynamic machinery. a, Needle wounding triggers mitochondrial fragmentation in *drp-1(tm1108)* mutant. Representative confocal images of epidermal mitochondria labeled with mito::GFP(*juEx4796*), before and after needle wounding (N = 10 independent experiment)). *drp-1* mutants showed extensively interconnected mitochondria in the epidermis before wounding. Mitochondria became fragmented after needle wounding in *drp-1* mutant. **b**, Epidermal mitochondrial morphology before and after Micropoint UV laser wounding in both WT and *drp-1(tm1108)* mutant (see also Supplementary Movie 5). mitochondrial were labeled by *mito::dendra2(juSi271)*. **c**, Quantitation of the farthest fragmented mitochondrial induced by laser wounding to the injury site (WT, n = 23; *drp-1*, n = 24 animals). We measured 20 distance from the wound site to the farthest fragmented left and right side and averaged them. Note, wounding induced mitochondrial fragmentation farther in *drp-1* mutant than in WT. Bars indicate mean ± SEM, ****, P < 0.0001, Two-tailed unpaired *t*-test. Source data are provided as a Source Data file. **d**, Laser wounding triggers mitochondrial fragmentation independent on mitochondrial fusion protein FZO-1 and EAT-3. Representative confocal images of mitochondria (labeled with P*col-19-mito::GFP(juEx4796)*) in WT and mitochondrial RNAi or mutants both before and 10 s after laser wounding (N = 3 independent experiment). *eat-3* RNAi was performed from the L1 stage. All wounding was performed at the young adult stage. **e**, Representative confocal images of epidermal mitochondria morphology and DRP-1 protein before and after Micropoint UV laser wounding (N = 2 independent experiment). P*col-19-mito::GFP; Pcol-19-*

mKate2::drp-1(zjuEx5) stain was used for imaging. **f-h**, Mitochondria were labeled by P*col-19-mito::GFP(juEx4796).* Representative confocal images of epidermal mitochondria morphology before and after needle wounding in mitochondrial fission factor *mff-1* and *mff-2* RNAi animals (**f**). *mff-1* and *mff-2* are two mitochondrial fission factor homologs; their functions in mitochondrial dynamics in *C. elegans* have not been extensively characterized. Mitochondrial morphology was also affected by other mitochondrial dynamics regulators, including CHCH-3, MOMA-1, LETM-1, and NCX-6. However, wounding still induces mitochondrial fragmentation in *chch-3(tm2336)*, *moma-1(tm1912)*, *letm-1(RNAi)*, and *ncx-6(RNAi)* animals (**g**). Wounding triggers mitochondrial fragmentation in BCL-2 family gene mutants (**h**). In *C. elegans*, apoptosis inhibitor Bcl2 family members CED-9 protein promotes mitochondrial fusion and its antagonist EGL-1 is capable of suppressing this activity [1-3]. However, loss or gain of function mutations in *egl-1(n487 or n1084;n3082)*, or gain of function in *ced-9(n1950sd)*, did not overtly affect epidermal mitochondrial morphology before wounding and mitochondria still became fragmented after needle wounding. Scale bar: (**a**-**h**): 10 μm. Red asterisks (**a**-**h**) indicate the wound site. N = 3 independent experiments.

Supplementary Figure 4

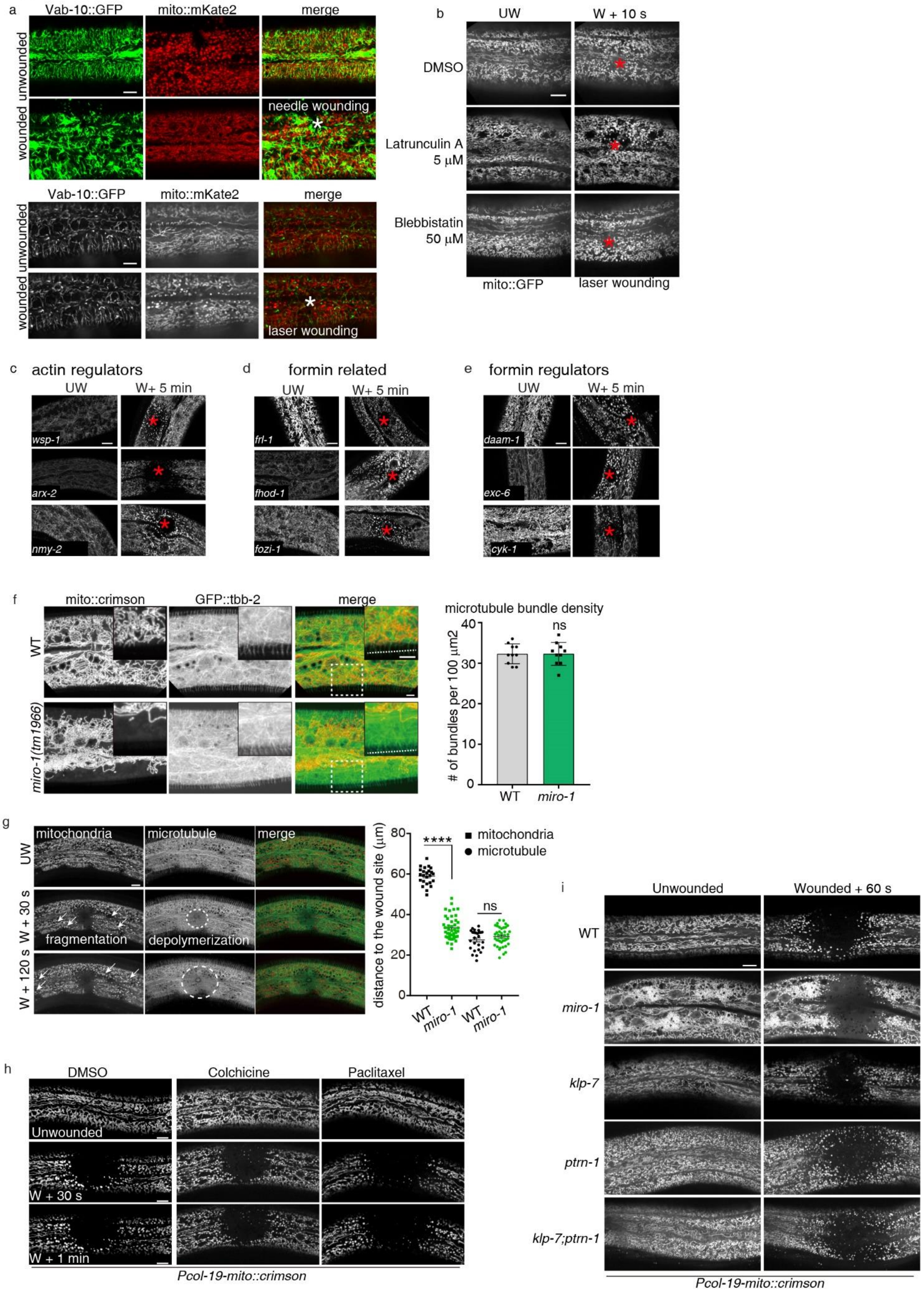

Supplementary Figure 4: WIMF is not dependent on cytoskeleton dynamics or formin related proteins.

Actin polymerization and inverted formin proteins (INF2) regulate mitochondrial fission and fusion [4, 5]. We labeled stabilized F-actin (vab-10$_{ABD}$::GFP) and mitochondria (mito::mKate2) in the epidermis and found wounding does not destroy stabilized F-actin morphology in the process of mitochondrial fragmentation (**a**). Latrunculin A and Blebbistatin, inhibitors of actin polymerization and non-muscle myosin, do not affect mitochondrial morphology before and after wounding compared to the control (**b**). Wounding also triggers mitochondrial fragmentation in actin regulator mutants, including actin polymerization factor WSP-1, ARX-2, non-muscle myosin NMY-1/2 (**c**), formin regulators FRL-1, FHOD-1, FOZI-1(**d**), and formin related genes DAAM-1, EXC-6, CYK-1(**e**). These results suggest that actin dynamics and formin regulators are not required for WIMF.

a, Representative confocal images of epidermal mitochondrial morphology (labeled with *mito::mKate2(juSi47)*) and actin (label with *Pcol-10-vab-10 ABD::GFP(juEx4284)*) before and after laser wounding. N = 3 independent experiments. Note, wounding does not affect *vab-10* $_{ABD}$*::GFP* expression pattern. **b**, Laser wounding triggers mitochondrial fragmentation in the animals treated with actin polymerization and myosin II inhibitors. Animals were cultured on Latrunculin A (5 μM), and Blebbistatin (50 μM) plates from the L4 stage and were subjected to laser wounding at the young adult stage. DMSO plates set as control. N = 3 independent experiments. **c**, Representative confocal images of epidermal mitochondrial morphology before and after needle wounding in actin regulators *wsp-1*, *arx-2,* and *nmy-2* RNAi knockdown animals. N = 2 independent experiments. **d**, Representative confocal images of epidermal mitochondria morphology before and after needle wounding in formin related protein RNAi animals, including *frl-1*, *fhod-1*, and *fozi-1*. N = 2 independent experiments. **e**, Representative confocal images of epidermal mitochondria morphology before and after needle wounding in *daam-1*, *exc-6,* and *cyk-1* RNAi animals. Scale bar (**a**-**e**): 10 μm. N = 2 independent experiments. **f**, Left, representative confocal image of microtubule and mitochondria in wild type and *miro-1(tm1966)* mutant (see also Supplementary Movie 8). N = 3 independent experiments. Mitochondria is labeled by P*col-19-mito::crimson(juIs492)*, microtubule is labeled by P*col-19-GFP::tbb-2(juIs492)*. The white dashed line indicates the quantitation area, Scale bar, 10 μm. White dotted squares indicate the zoom-in area, Scale bar in an enlarged image, 2 μm. Right, quantitation of microtubule bundle density in the wild type (n = 10) and *miro-1(tm1966)* mutant (n = 10). Bars indicate mean ± SEM. ns, P > 0.9999, Two-tailed unpaired *t*-test. **g**, Left, representative confocal image of mitochondrial fragmentation and microtubule depolymerization after Micropoint UV laser injury (see also Supplementary Movie 9 and Movie 10). N = 5 independent experiments. A white dotted circle indicates the microtubule depolymerization area, and the white arrow indicates fragmented mitochondria. Note, microtubule depolymerization area is smaller than the mitochondrial fragmentation area and has no significant change in *miro-1(tm1966)* mutant. Scale bar: 10 μm. Right, quantitation of the distance of either mitochondrial fragmentation or microtubule depolymerization after wounding (WT-mito, n = 24; WT-MT, n = 26; *miro-1*-mito, n = 43; *miro-1*-MT, n = 40 animals). Bars indicate mean ± SEM. ns, P = 0.1794, ****, P < 0.0001, Two-tailed unpaired *t*-test. Source data are provided as a Source Data file. **h**, Representative confocal images of mitochondria morphology with the drug treatment before and after wounding. N = 2 independent experiments. Colchicine inhibits microtubule polymerization, while paclitaxel stabilizes the microtubule. Neither of these drugs treatment affects WIMF. **i**, WIMF is not dependent on microtubule dynamic or its motor proteins. N = 2 independent experiments. Mitochondrial morphology in *miro-1(tm1966), klp-7(tm2143), ptrn-1(lt1)* mutants

before and after wounding. Mitochondria is labeled by P*col-19-mito::crimson(juIs492)* (**h,i**). Scale bar, 10 μm.

Supplementary Figure 5

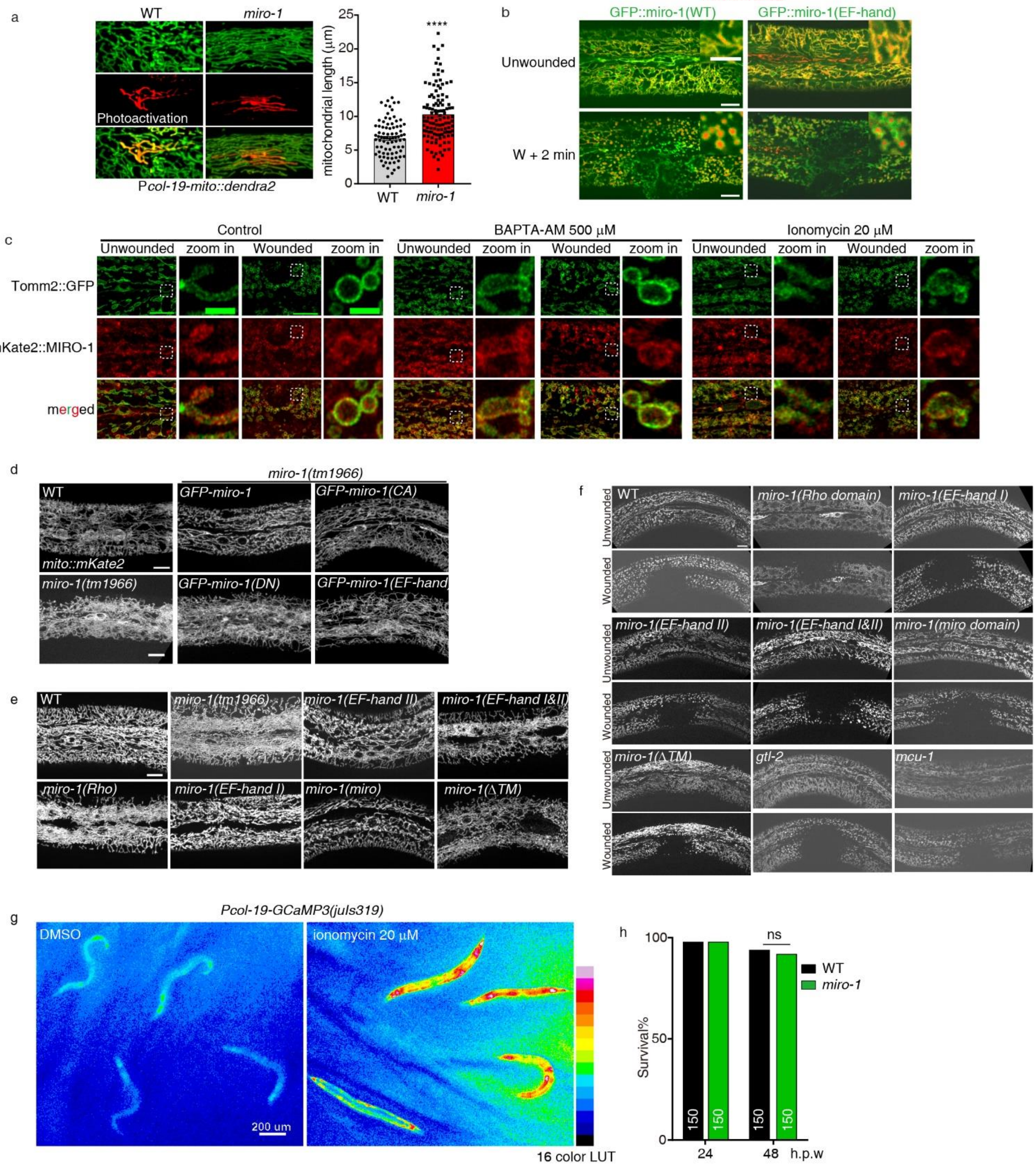

Supplementary Figure 5: WIMF is dependent on mitochondrial Rho GTPase MIRO-1 and cytosolic Ca^{2+} signals. a, Left, representative confocal image of P*col-19-mito::dendra2(juSi271)* after photoconversion. WT mitochondria form a network that is disrupted in *miro-1(tm1966)* mutant. Scale bar: 2 μm. Right, quantitation of mitochondrion length in WT (n = 48) and *miro-1* mutant (n = 51) before and after wounding. Bars indicate mean ± SEM. ****, P < 0.0001, Two-tailed unpaired *t*-test. **b**, confocal images of GFP::MIRO-1 and GFP::MIRO-1(ΔEF-hand) with mitochondria (labeled by mito::mKate2) before and 2 mins after laser wounding (see also Supplementary Movie 6). N = 3 independent experiments. Note, both GFP::MIRO-1 and GFP::MIRO-1(ΔEF-hand) localized to the outer

side of the mito::mKate2. Scale bar, 10 μm, 5 μm (zoom-in image). **c**, confocal images of mKate2::MIRO-1 and Tomm-20::GFP before and after wounding in either DMSO, BAPTA-AM, and ionomycin conditions. N = 3 independent experiments. The strain (*mKate2::miro-1(zju211);Tomm-20::GFP(zju201)*) were treated with BAPTA-AM (500 μM) or ionomycin (10 μM) for 3 hours and then performed laser wounding. Note, mKate2::MIRO-1 and Tomm-20::GFP partially colocalized before and after wounding, suggesting MIRO-1 is localized to the outer mitochondrial membrane. Scale bar: 10 μm, 5 μm (zoom-in image). **d,** Representative confocal images of mitochondria (labeled by P*col-19-mito::mKate2(zjuSi47))* in the *miro-1(tm1966)* mutant and transgenic *GFP::miro-1* background. N = 2 independent experiments. Note, P*col-19-GFP::miro-1(zjuEx57),* P*col-19-GFP::miro-1(CA)(zjuEx63),* but not P*col-19-GFP::miro-1(EF-hand)*(*zjuEx59)* and P*col-19-GFP::miro-1(DN)(zjuEx73)* rescue the morphology of mitochondria.CA: constitutively active, DN: dominant-negative, EF-hand: EF-hand mutation. Scale bar: 10 μm. **e**, Representative confocal images of mitochondria in wild type and *miro-1* mutants. N = 5 independent experiments. Domain-specific mutations were generated using the CRISPR-Cas9 system. *miro-1(ΔRho)(zju44)*, *miro-1(EF-hand II)(zju162),* and *miro-1(ΔTM)(zju87)* show similar mitochondrial morphology as *miro-1(tm1966)*, suggesting these mutations disrupt the function of MIRO-1. Scale bars: 10 μm. **f**, Representative confocal images of epidermal mitochondria before and after Micropoint UV laser wounding in WT *miro-1* mutants including ΔRho-1 *miro-1(zju44),* Δ EF-hand I *miro-1*(*zju75),* ΔEF-hand II *miro-1(zju162),* ΔEF-hand I&II *miro-1(zju75;zju162),* ΔMIRO *miro-1(zju84),* ΔTM *miro-1(zju87), gtl-2(n2618),* and *mcu-1(ju1154)* mutants (see also Supplementary Movie 11 and Movie 12). N = 3 independent experiments. Rho, EF-hand II, and Transmembrane domain but not EF-hand I and MIRO domain are required for response to the injury. GTL-2 but not MCU-1 is required for mitochondrial fragmentation after wounding. Scale bar:10 μm. **g,** Ionomycin treatment increase the cytosolic Ca^{2+} level. Epidermal specific Ca^{2+} sensor strain *Pcol-19-GCaMP3(juIs319)* were treated with 20 μM ionomycin for 3 hours, DMSO treatment as control. N = 2 independent experiments. Images were processed by image J and shown in 16-color LUT. Scale, 200 μm. **h**, 24 h, and 48 h post-wounding survival in both WT and miro-1 mutant. We wounded animals twice in the anterior and posterior body and checked their survival 24 h and 48 h later. The survival of wounded animals was normalized to unwounded animals. N = 150 animals. Two-sided Fisher's exact test. Source data are provided as a Source Data file.

Supplementary Figure 6

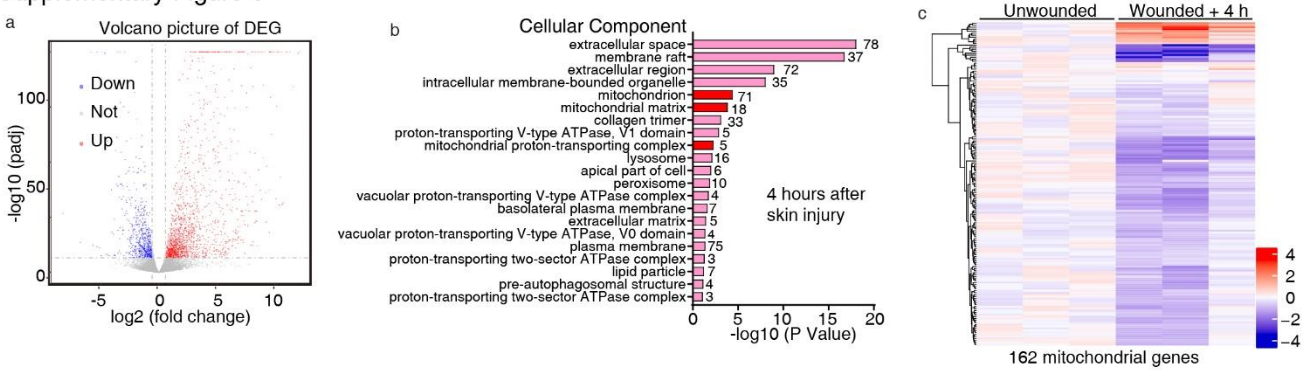

d

Gene Name	Gene Description	Human Homology
acdh-2	Acyl CoA deHydrogenase	ALDH2
cyp-13A8	CYtochrome P450 family	Cytochrome P450 3A4 isoform 2
cyp-13A12	CYtochrome P450 family	Cytochrome P450 3A4
cyp-14A4	CYtochrome P450 family	Cytochrome P450 2C8
cyp-32B1	CYtochrome P450 family	CYP19A1， and CYP4V2
cyp-33C8	CYtochrome P450 family	CYP2R1
cyp-33D3	CYtochrome P450 family	CYP2J2
cyp-35A2	CYtochrome P450 family	CYP2J2
fat-6	Delta(9)-fatty-acid desaturase fat-6	Acyl-CoA desaturase
dhs-23	DeHydrogenases, Short chain	NA
dhs-25	DeHydrogenases, Short chain	NA
dpyd-1	Dihydropyrimidine dehydrogenase [NADP(+)]	DPYD
drd-1	dietary restriction down regulated	FAXDC2
gst-20	Glutathione S-Transferase	HPGDS
ldh-1	L-lactate dehydrogenase	LDHB
sod-3	Superoxide dismutase [Mn] 2, mitochondrial	superoxide dismutase
F12E12.11	hypothetical protein	α−aminoadipate semialdehyde synthase (AASS)
R02D3.1	hypothetical protein	HSD17B14
R05D8.9	hypothetical protein	HSD17B14

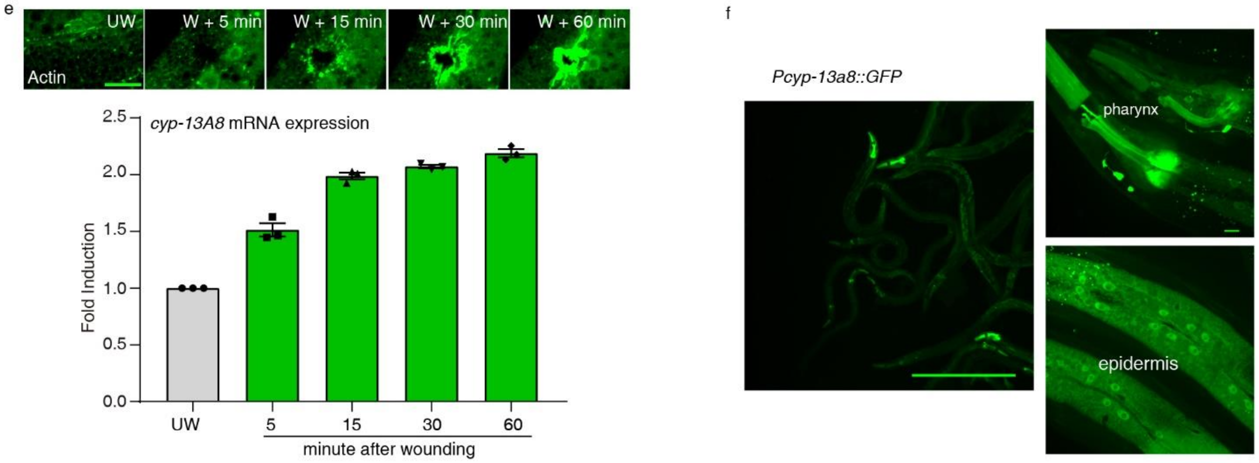

Supplementary Figure 6: Transcriptional regulation comparison in WT and *fzo-1* mutant. **a**, Volcano plot of differentiated expressed genes (DEGs) in wild type animals before and 4 hours after needle wounding, as determined by single worm RNA sequencing. Up-regulated, down-regulated, and no change genes are shown in red, blue, and gray, respectively. The horizontal dashed line shows the p-value cutoff at 0.05, while the two vertical dashed lines show the fold change cutoff at 1.5. The max value of y-axis is truncated at 20 for illustration. **b**, Representative top GO term category of cellular component analysis of DEGs induced 4 hours after wounding in *C. elegans* as described in a. Single worm RNA-seq was done on unwounded and wounded animals. Note, Mitochondrial specific GO terms are highlighted in red. **c**, a Heat map of 162 mitochondrial enriched genes from GO term cellular components after needle wounding. The gene name and true fold changes are in Supplementary Data 1. Fold change (FC) was calculated by comparing normalized count

values of each condition to WT unwounded control and then transformed to log2. Each row is one gene, and each column is one sample. **d**, List of upregulated genes involved in the oxidative-reduction process after GO term biological process analysis. **e**, Top, actin-ring formation at the wound site at different times after wounding. Scale bar: 10 μm. Bottom, qPCR detection of *cyp-13a8* mRNA expression level in animals with unwounded and different time points after wounding. Bars indicate mean ± SEM. N = 3 independent experiments. Source data are provided as a Source Data file. **f,** The expression pattern of *cyp-13A8*. A 2 kb promoter of *cyp-13A8* was cloned upstream of the first exon and fused with GFP sequence to make *Pcyp-13A8-GFP(zjuEx804)* strain. Scale bar: 10 μm. N = 2 independent experiments.

Supplementary Figure 7

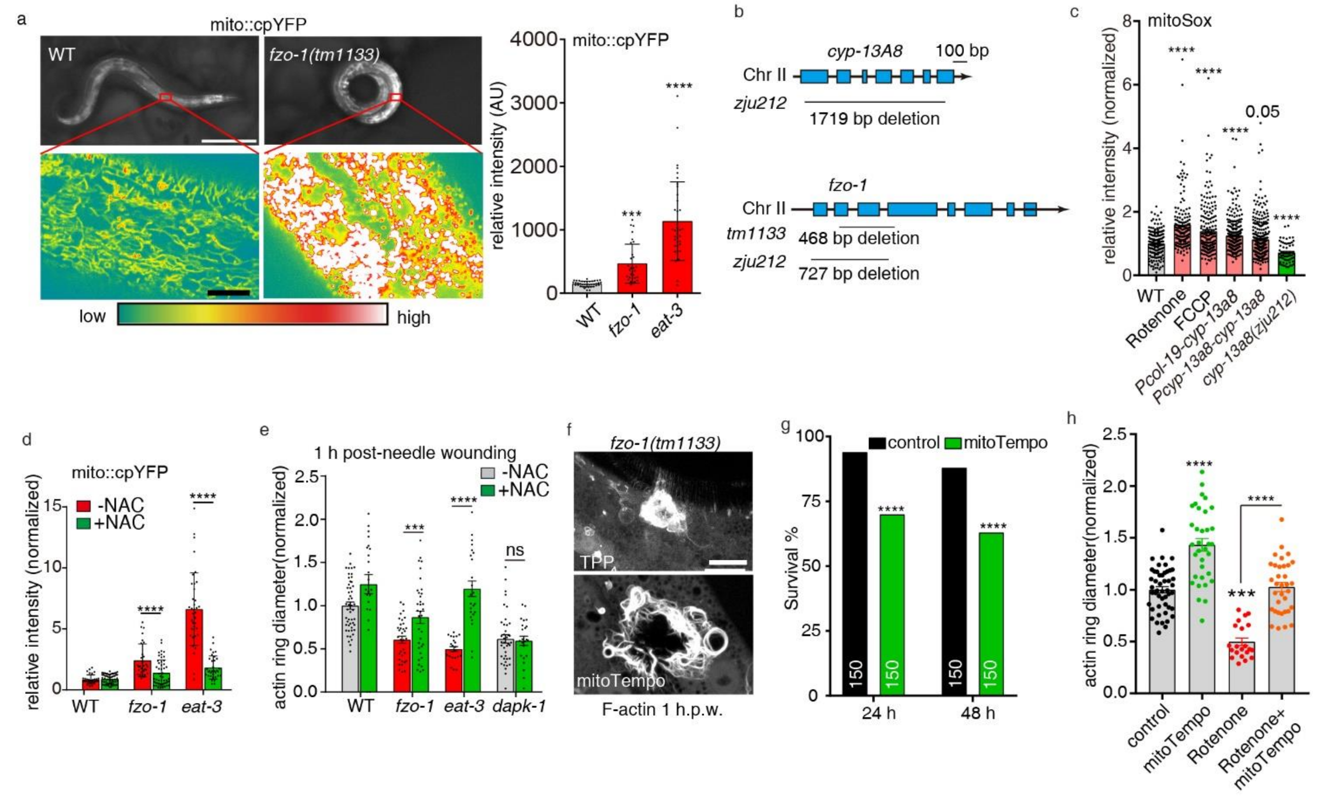

Supplementary Figure 7: Enhanced mitochondrial fragmentation accelerated wound closure is dependent on mtROS. a, Loss of function in *fzo-1* causes increased *mito::cpYFP(juEx5344)* fluorescence. Representative images of mito::cpYFP fluorescence signal in wild type and *fzo-1* mutants. Left Top: mito::cpYFP signal in whole animals; Scale bar: 250 μm. Left bottom: confocal images of epidermal mito::cpYFP in both WT and *fzo-1(tm1133)* mutant (intensity color code); Scale bar:10 μm. Right, quantitation of the average intensity of mito::cpYFP (WT, n = 16; *fzo-1*, n = 30; *eat-3*, n= 38 animals). Bars indicate mean ± SEM, ***, P = 0.0019, ****, P < 0.0001, versus WT. Two tailed unpaired *t*-test. **b**, diagram of genomic deletion in *fzo-1* and *cyp-13A8* genomic locus using CRISPR-Cas9 system. There are 1719 bp deletion *cyp-13A8(zju212)* and 727 bp deletion in *fzo-1* gene. The deletion size of *zju212* is larger than *tm1133*. **c**, Relative fluorescence intensity of mitoSox staining in WT animals (n = 170) treated FCCP (n = 200) and Rotenone (n = 157) or expressed with *cyp-13A8* transgene (n = 260) or *cyp-13a8* knock out animals (n = 620 mitochondria). Note, FCCP, Rotenone and overexpression of *cyp-13a8* increase the level of mitoSox staining while *cyp-13A8(zju212)* show reduced mitoSox staining. All the intensity were normalized to WT. Bars indicate mean ± SEM, ****, P < 0.000, (versus WT), One-way ANOVA Dunnett's test. **d**, Quantitation of mito::cpYFP intensity (WT, n = 32; NAC, n = 54; *fzo-1*, n = 30; *fzo-1*-NAC, n = 50; *eat-3*, n = 38; *eat-3*-NAC, n = 34 animals). L4 stage animals were treated with NAC (10 mM) overnight and mito::cpYFP fluorescence images were taken for intensity analysis. Bars indicate mean ± SEM. ****, P < 0.0001, Two-tailed unpaired *t*-test. **e**, The accelerated wound closure of mitochondrial mutants is dependent on mtROS. Quantitation of actin ring diameter in needle wounded worms (WT, n = 50; NAC, n = 23; *fzo-1*, n = 37; *fzo-1*-NAC, n = 42; *eat-3*, n = 23; *eat-3*-NAC, n = 24; *dapk-1*, n = 40; *dapk-1*-NAC, n = 23 animals). Bars indicate mean ± SEM. ns, P = 0.774, **, P = 0.0025, ****, P < 0.0001, versus control (no NAC), Two-tailed unpaired *t*-test. **f**, confocal images of actin ring formation 1 h.p.w in *fzo-1(tm1133)* mutant and treated with mitoTempo. Scale bar: 10 μm. N = 3 independent experiments. **g**, Quantitation of

post-wounding survival in the animals with mitoTempo treatment. mitoTempo significantly reduced the survival rate 24 and 48 h.p.w. N = 150，****, P < 0.0001, Two-sided Fisher's exact test. **h**, quantitation of actin ring diameter in the WT animals (n = 48) treated with mitoTempo (n = 34), Rotenone (n = 21) and their combination (n = 34) after needle wounding. Bars indicate mean ± SEM, ****, P < 0.0001, Two tailed unpaired *t*-test. Source data are provided as a Source Data file.

Fu et al., Supplementary Table 1

Strains that are used in this study

Strains/organisms	Source	Code
Cell line: U2OS cell line	ATCC	ATCC@HT B96
Zebrafish	ZJU	WT AB line
C.elegans: CU6372(*drp-1(tm1108)*IV)	CGC	CU6372
C.elegans: CU5991(*fzo-1(tm1133)*II)	CGC	CU5991
C.elegans: CZ9957(*gtl-2(n2618)*IV)	CGC	CZ9957
C.elegans: CZ17124(*eat-3(tm1107)*II)	Chisholm lab	CZ17124
C.elegans: FX01966(*miro-1(tm1966)*IV)	CGC	FX01966
C.elegans: CZ14748(P*col-19*-GFP::moesin*(juIs352)*I*)*	*Xu and Chisholm, 2011*[6]	CZ14748
C.elegans: CZ14758(*gtl-2(n2618)*IV*;*P*col-19*-GFP::moesin*(juIs352)*I)	*Xu and Chisholm, 2011*[6]	CZ14758
C.elegans: CZ16518(P*col-19*-mito::GFP*(juEx4796)*)	This study	CZ16518
C.elegans: CZ17086(*drp-1(tm1108)*IV*;*P*col-19*-GFP::moesin*(juIs352)*I)	This study	CZ17086
C.elegans: CZ17087(*fzo-1(tm1133)*II*;*P*col-19*-GFP::moesin*(juIs352)*I)	This study	CZ17087
C.elegans: CZ17906(*eat-3(tm1107)*II*;*P*col-19*-GFP::moesin*(juIs352)*I)	This study	CZ17906
C.elegans: CZ19100(P*col-19*-GFP::moesin*(juIs352)*I*;fzo-1(tm1133)*II*;gtl-2(n2618)*IV)	This study	CZ19100
C.elegans: CZ20105(*mcu-1(ju1154)*IV*;*P*col-19*-GFP::moesin*(juIs352)*I)	*Xu and Chisholm, 2014* [7]	CZ20105
C.elegans: CZ21768(*fzo-1(tm1133)*II*;mcu-1(ju1154)*IV*;*P*col-19*-GFP::moesin*(juIs352)*I)	This study	CZ21768
C.elegans: CZ22411(*miro-1(tm1966)*IV*;*P*col-19*-mito::GFP*(juEx4796)*)	This study	CZ22411
C.elegans: CZ22488(P*col-19-mito::dendra2(juSi271)*I)	This study	CZ22488
C.elegans: CZ6326(P*nlp-29*-GFP*(frIs7)*IV)	*Pujol et al., 2008* [8, 9]	CZ6326
C.elegans: SHX10 (P*hyp-7*-mito::GFP*(yqIs157)* V)	Zhou et al., 2018[9]	SHX10
C.elegans: SHX29(P*col-19*-mKate::DRP-1*;*P*col-19*-mito::GFP*(zjuEx9)*)	This study	SHX29
C.elegans: SHX51(P*col-19*-GFP::tbb-2;P*col-19*-mito::Crimson(*juIs491*);*klp-7(tm2143)*III)	This study	SHX51
C.elegans: SHX52(P*col-19*-GFP::tbb-2;P*col-19*-mito::Crimson(*juIs492*);*ptrn-1(lt1)*X)	This study	SHX52
C.elegans: SHX53(P*col-19*-GFP::tbb-2;P*col-19*-mito::Crimson(*juIs491*);*ptrn-1(lt2)*X;*klp-7(tm2143)*III)	This study	SHX53
C.elegans: SHX63(*gfp::miro-1(zju21)*IV)	This study	SHX63
C.elegans: SHX104(*miro-1(tm1966)*IV*;*P*col-19*-GFP::moesin*(juIs352)*I)	This study	SHX104

C.elegans: SHX114(*fzo-1(tm1133)*II;*miro-1(tm1966)*IV;P*col-19*-GFP::moesin*(juIs352)*I)	This study	SHX114
C.elegans: SHX143(*miro-1(zju44)* IV;P*hpy-7*-mito::GFP(yqIs157)V)	This study	SHX143
C.elegans: SHX187(*miro-1(zju30)*IV;P*hyp-7*-mito::GFP*(yqIs157)*V)	This study	SHX187
C.elegans: SHX210(*miro-1(zju87)*IV;P*hyp-7*-mito::GFP*(yqIs157)*V)	This study	SHX210
C.elegans: SHX214(P*col-19*-GFP::moesin*(juIs352)*I;*fzo-1(tm1133)*II)	This study	SHX214
C.elegans: SHX231(P*col-19*-lifeact::PHtomato*(zjuSi22)*I)	This study	SHX231
C.elegans: SHX264(*miro-1(zju75)*IV;P*hyp-7*-mito::GFP*(yqIs157)*V)	This study	SHX264
C.elegans: SHX324(*fzo-1::gfp(zju136)*II)	This study	SHX324
C.elegans: SHX365(P*col-19-GFP::miro-1(cDNA)(EF-hand mutation E212K+E332K);*P*col-19*-mito::mKate2(*zjuEx59);miro-1(tm1966)*IV)	This study	SHX365
C.elegans: SHX366(P*col-19-GFP::miro-1(cDNA)(Constitute active E18V+K435V);*P*col-19*-mito::mKate2*(zjuEx63); miro-1(tm1966)*IV)	This study	SHX366
C.elegans: SHX367(P*col-19-GFP::miro-1(cDNA)(Dominant negative T23N+T440N);*P*col-19*-mito::mKate2*(zjuEx73);miro-1(tm1966)*IV)	This study	SHX367
C.elegans: SHX373(P*col-19-GFP::miro-1(genome)*;P*col-19*-mito::mKate2*(zjuEx57);miro-1(tm1966)*IV)	This study	SHX373
C.elegans: SHX377(P*col-19*-mito::mKate2*(zjuSi47)*II)	This study	SHX377
C.elegans: CZ12680(P*col-19*-eGFP::rGBD(*juEx3025*)	Xu and Chisholm, 2014 [7]	CZ12680
C.elegans: CZ17255(P*col-19*-eGFP::rGBD(*juEx3025*);*drp-1(tm1108)*IV)	This study	CZ17255
C.elegans: CZ17256(*Pcol-19*-eGFP::rGBD*(juEx3025)*;*fzo-1(tm1133)*II)	This study	CZ17256
*C.elegans:*CZ17257(P*col-19*-eGFP::rGBD*(juEx3025);eat-3(tm1107)*II)	This study	CZ17257
C.elegans: SHX447(*miro-1(zju75)*IV;P*hyp-7*-mito::GFP(*yqIs157*)V;*miro-1(zju162)*IV)	This study	SHX447
C.elegans: SHX471(P*col-19*-lifeact::PHtomato*(zjuSi22)*I;*fzo-1::gfp(zju136)*II)	This study	SHX471
C.elegans: SHX483(*fzo-1(tm1133)*II;P*col-19*-mito::dendra2*(juSi271)*I)	This study	SHX483
C.elegans: SHX522(*fzo-1(tm1133)*II;P*col-19*-mito::dendra2*(juSi271)*I;*phsp-16.2*-FZO-1::tag-RFP-T*(zjuEx49)*)	This study	SHX522
C.elegans: SHX537(P*col-19-vhhgfp-zif1(zjuEx99);*P*col-19*-lifeact::PHtomato*(zjuSi22)*I;*fzo-1::gfp(zju136)*II)	This study	SHX537
C.elegans: SHX567(P*col-19*-lifeact::PHtomato*(zjuSi22)*I; P*col-19-vhhgfp-zif1(zjuEx99)*)	This study	SHX567

C.elegans: SHX568*fzo-1::gfp(zju136)*IIP*col-19-vhhgfp-zif 1(zjuEx99)*)	This study	SHX568
C.elegans: SHX616(*drp-1(tm1108)* IV;P*col-19*-mito::dendra2*(juSi271)*I)	This study	SHX616
C.elegans: SHX633(*fzo-1(tm1133)*II;*miro-1(tm1966)*IV;P*col-19*-Tomm-20::GFP*(zjuSi48)*I)	This study	SHX633
C.elegans: SHX666(*fzo-1(tm1133)*II;*drp-1(tm1108)*IV;P*col-19*-mito::dendra2*(juSi271)*I)	This study	SHX666
C.elegans: SHX667(P*myo-3-vhhgfp-zif1(zjuEx154);*P*col-19*-lifeact::PHtomato*(zjuSi22)*I;*fzo::gfp(zju136)*II)	This study	SHX667
C.elegans: SHX668(*fzo-1(tm1133)*II;P*col-19*-mito::dendra2*(juSi271)*I;P*sur-5-fzo-1(cDNA)(zjuEx165)*)	This study	SHX668
C.elegans: SHX722(P*myo-3-vhhgfp-zif1(zjuEx158);*P*col-19*-lifeact::PHtomato*(zjuSi22)*I)	This study	SHX722
C.elegans: SHX736(*miro-1(zju75)*IV;P*col-19*-mito::dendra2*(juSi271)*I)	This study	SHX736
C.elegans: SHX738(*miro-1(zju75)*IV;*miro-1(zju162)*IV;P*col-19*-mito::dendra2(*juSi271)*I)	This study	SHX738
C.elegans: SHX739(P*col-19*-mito::mKate2*(zjuSi47)*II;*gfp::miro-1(zju21)*)	This study	SHX739
C.elegans: SHX772(*miro-1(zju44)*IV;P*col-19*-mito::dendra2*(juSi271)*I)	This study	SHX772
C.elegans: SHX780(*miro-1(zju30)*IV;P*col-19*-mito::dendra2*(juSi271)*I)	This study	SHX780
C.elegans: SHX819(P*col-19*-lifeact::PHtomato*(zjuSi22)*I*fzo-1::gfp(zju136)*II;P*sur-5-vhhgfp-zif1(zjuEx220)*)	This study	SHX819
C.elegans: SHX821(P*col-19*-lifeact::PHtomato*(zjuSi22)*I*;* P*sur-5-vhhgfp-zif1*)	This study	SHX821
C.elegans: SHX840(*miro-1(zju87)*IV;P*col-19*-mito::dendra2*(juSi271)*I)	This study	SHX840
C.elegans: SHX881(P*col-19*-mito::mKate2*(zjuSi47)*II;*miro-1(tm1966)*IV)	This study	SHX881
C.elegans: SHX891(*gtl-2(n2618)*IV;P*col-19*-mito::dendra2*(juSi271)*I)	This study	SHX891
C.elegans: SHX892(*miro-1(zju162)*IV;P*col-19*-mito::dendra2*(juSi271)*I)	This study	SHX892
C.elegans: SHX894(*fzo-1(tm1133)*II;P*col-19*-GFP::moesin*(juIs352)*I;P*fzo-1*-FZO-1::mkate2*(zjuEx230)*)	This study	SHX894
C.elegans: SHX895(*fzo-1(tm1133)*II;P*col-19*-GFP::moesin*(juIs352)*I;P*hsp-16.2-fzo-1*-RFP-T*(zjuEx228)*)	This study	SHX895
C.elegans: SHX896(*fzo-1(tm1133)*II;P*col-19*-GFP::moesin*(juIs352)*I;P*dpy-7-fzo-1(zjuEx232)*)	This study	SHX896
C.elegans: SHX897(*fzo-1(tm1133)*II;P*col-19-GFP::moesin(juIs352)*I;P*myo-3-fzo-1(cDNA)(zjuEx238)*)	This study	SHX897
C.elegans: SHX912(P*col-19*-mito::dendra2*(juSi271)* I*; mcu-1(ju1154)* IV)	This study	SHX912

C.elegans: SHX927(*miro-1(zju40)*IV;P*col-19*-mito::dendra2*(juSi271)*I;*mcu-1(ju1154)*IV)	This study	SHX927
C.elegans: SHX928(*miro-1(zju19)*IV;*gtl-2(n2618)*IV;P*col-19*-mito::dendra2*(juSi271)*I)	This study	SHX928
C.elegans: SHX1231(*miro-1(tm1966))*IV;P*hyp-7*-mito::GFP*(yqIs157*)V)	This study	SHX1231
C.elegans: SHX1488(*cyp-13a8(zju212)*II)	This study	SHX1488
C.elegans: SHX1603(P*col-19*-GFP::moesin(*juIs352)*I;*cyp-13a8(zju212)*II)	This study	SHX1603
C.elegans: SHX1611(P*col-19*-GFP::moesin(*juIs352)*I;P*col-19*-mito-mKate2(*zjuSi47*)II)	This study	SHX1611
C.elegans: SHX1692(P*col-19*-CYP-13A8(*zjuEx786*))	This study	SHX1292
C.elegans: SHX1698(P*cyp-13a8*-CYP-13A8(*zjuEx792))*	This study	SHX1698
C.elegans: SHX1714(mKate2::MIRO-1(*zju211*)IV;TOMM-20::GFP(*zju201*)V	This study	SHX1714
C.elegans: SHX1720(P*cyp-13a8*-GFP(*zjuEx804*))	This study	SHX1720

Fu et al., Supplementary Table 2

Plasmids that are used in this study

**Plasmids**	**Source**	**Code**
pSX269(P*col-19*-mito::GFP)	This study	pSX269
pSX280(P*col-19*-Tomm20 N55::GFP)	This study	pSX280
pSX480(P*drp-1-drp-1*::mKate2)	This study	pSX480
pSX485(P*fzo-1-loxp-fzo-1-loxp-mKate2*)	This study	pSX485
pSX519(P*col-19-mito::mKate2*)	This study	pSX519
pSX533(pCFJ210-FRT-HygR-FRT-*Pcol*-19-mito::Dendra2)	This study	pSX533
pSX696(PU6-sgRNA-*miro-1*)	This study	pSX696
pSX697(*gfp*::*miro-1* SEC repair template)	This study	pSX697
pSX707(PU6-sgRNA-*miro-1* on EF-Hand I Domain)	This study	pSX707
pSX708(PU6-sgRNA-*miro-1* on Miro Domain)	This study	pSX708
pSX709(PU6-sgRNA-*miro-1* on Rho Domain)	This study	pSX709
pSX710(PU6-sgRNA-*miro-1* on Trans-Membrane Domain)	This study	pSX710
pSX711(pCR8-*miro-1*(Genome))	This study	pSX711
pSX753(pCR8-*miro-1*(cDNA) (Constitute Active E18V+K435V))	This study	pSX753
pSX755(pCR8-*miro-1*(cDNA) (EF-Hand Mutation E212K+E332K))	This study	pSX755
pSX757(pCR8-*miro-1*(cDNA) (Dominant Negative T23N+T440N))	This study	pSX757
pSX765(P*hsp-16.2-fzo-1*::TagRFP)	This study	pSX765
pSX771(P*eft-3*-Cas9+*fzo-1* sgRNA #159)	This study	pSX771
pSX780(*fzo-1*::*gfp* SEC repair template)	This study	pSX780
pSX802(P*col-19-gfp*::*miro-1*(cDNA) (EF-Hand Mutation E212K+E332K))	This study	pSX802
pSX803(P*col-19-gfp::miro-1*(cDNA) (Constitute Active E18V+K435V)	This study	pSX803
pSX804(P*col-19-gfp::miro-1*(cDNA) (Dominant Negative T23N+T440N)	This study	pSX804
pSX805(P*col-19-gfp::miro-1*(Genome))	This study	pSX805
pSX822(*fzo-1::gfp* repair template #1)	This study	pSX822
pSX823(PU6-sgRNA-*miro-1* on EF-Hand II Domain)	This study	pSX823
pSX838(*fzo-1::gfp* repair template #2)	This study	pSX838
pSX873(pCR8-*Pcol-19*-mito::mKate2)	This study	pSX873
pSX881(P*col-19-vhhgfp::zif-1*)	This study	pSX881
pSX976(P*myo-3-fzo-1*(cDNA))	This study	pSX976
pSX977(P*dpy-7-fzo-1*(cDNA))	This study	pSX977
pSX978(P*sur-5-fzo-1*(cDNA))	This study	pSX978
pSX985(P*myo-3-vhhgfp::zif-1*)	This study	pSX985
pSX1129(P*sur-5-vhhgfp::zif-1*)	This study	pSX1129
pSX1676(Pcyp-13a8-GFP)	This study	pSX1676
pSX1678(Pcol-19-CYP-13A8)	This study	pSX1678
pSX1681(Pcyp-13a8-CYP-13A8)	This study	pSX1681

Fu et al., Supplementary Table 3

Primers that are used in this study

Primers	Gene	sequences
zju902	act-1 forward	CCAGGAATTGCTGATCGTATGC
zju903	act-1 reverse	CGATCCAGACGGAGTACTTGC
zju1723	cyp-35A3 forward	GATTTGTGGTTAACCGGACAAG
zju1724	cyp-35A3 reverse	GATGAACGGTCAGTAAGACTG
zju1727	cyp-35D1 forward	CATGGATCACGTGATGAAGGG
zju1728	cyp-35D1 reverse	ATGTTGGCCACCAGCAATC
zju1729	gpx-6 forward	GATGGAATCTACAGGGAAAAAG
zju1730	gpx-6 reverse	GTGTTCTGACCATTTACCTCGATC
zju1799	sod-3 for	TCGGTTCCCTGGATAACTTG
zju1800	sod-3 rev	AAAGTGGGACCATTCCTTCC
zju1807	cyp-35A1 forward	GGTGCTGCGTTTGAAGTCTTC
zju1808	cyp-35A1 reverse	CTGCCGCTGCAAATCTTTTAGC
zju1811	dhs-28 forward	TCCTCAAGATGACCGAGCTT
zju1812	dhs-28 reverse	CTTGGGCGAGAGAGTTTGAC
zju1827	cyp-13A12 forward	TCGTGGAATAGCTGGACCAT
zju1828	cyp-13A12 reverse	ACTTCCTGCACCAAATCAGG
zju1829	F12E12.11 forward	GAAGTGTGGTGACGTTGGTG
zju1830	F12E12.11 reverse	ACGGACTCCGTGTTGAATC
zju1831	dhs-23 forward	AACTCGGTTAGCCCTGGAGT
zju1832	dhs-23 reverse	TGCCCAATAATGTACGACGA
zju1833	cyp-32B1 forward	GCCAGCCGGAATAAACTGTA
zju1834	cyp-32B1 reverse	AATGGGACGAATGCGAATGG
zju1835	gst-20 forward	ACGAGGATATCCGCATTGAG
zju1836	gst-20 reverse	CCGCGTAGAAATCCTTGAAC
zju1837	idh-1 forward	TTGTGCCTTGTTGATGTGGT
zju1838	idh-1 reverse	GTTGTGGGATGATTCCCTTG
zju1861	cyp-13A8 forward	CCTTGGGGAGTTCCGATTTTC
zju1862	cyp-13A8 reverse	CCATGACTTTCTGCATTCCTTCA
zju1863	cyp-14A4 forward	TCTATGGGCCATGCTACAC
zju1864	cyp-14A4 reverse	CGTGAACATGGAGCAATGAC
zju1865	cyp-33C8 forward	CCTACAGACTAACGGGCATG
zju1866	cyp-33C8 reverse	CATCCTGCTCCTCGCCAATAT
zju1867	cyp-33D3 forward	CTGGACACAAATGGGCATATGT
zju1868	cyp-33D3 reverse	CTCCATGTGCATCGAGTTCC
zju1869	dhs-25 forward	AATTGCTGTGGTTACCGGT
zju1870	dhs-25 reverse	CCTTCACACTGTCTGCATTTG
zju1871	dpyd-1 forward	TTGCCCAACCCAATTGGATG

zju1872	dpyd-1 reverse	GATAGCGCCTTCTTCACTTGC
zju1873	drd-1 forward	CGGAAGTCATGTGATTACTCTCTG
zju1874	drd-1 reverse	GGTAGTCATGTGGTTCTGGAG
zju1875	fat-6 forward	TGCAGCCATCGGACTCTAC
zju1876	fat-6 reverse	CGGCGGTTATTCCAAATCCT
zju41	eat-3 for	CGATTCGTCATACAACACC
zju42	eat-3 rev	GGAAATAGCTTTCCTTCAAG
zju47	eat-3 int for	CTGTGAAAGGTCCGAATCTC
zju45	drp-1 for	ACAGAGTTGTCTCCCTCTC
zju46	drp-1 rev	CAATGGATTGACAGATTCC
zju49	drp-1 int for	GACAACAAACTGATCGTGGA
zju742	fzo-1 for	CGTTGTCTCCCGATAATC
zju743	fzo-1 rev	GCACGCTATTCAGATTGAG
zju431	fzo-1 int for	GGGACATACAACATGTTGC
zju181	miro-1 for	AATTTATCGAACCAGGTCCG
zju225	miro-1 rev	ATTTATCAATCCCGCCTCCG
zju404	miro-1 int for	TGCGGAAGTTCGGTTATGAGA
zju405	miro-1 int rev	AGATCCTTCTTCCGTTCACGA
zju234	miro-1 mutations test for	CGGGTTTCCAGTCGGAAG
zju235	miro-1 mutations test rev	ATAATTACAGTCGACCCGGC
zju264	E18V For	CGGCGACGtcGGATGCGGAAAGAC
zju265	E18V Rev	ATCAGAACGATTCGGACGTC
zju266	T23N For	GAAAGAacTCGCTGGTGATGAG
zju267	T23N Rev	CGCATCCTTCGTCGCCGATC
zju268	E212K For	GATACAaAGCTCAACGATTTCCAG
zju269	E212K Rev	ACTGAGATATCCATCGTTGTCTCG
zju270	K435V For	GAGCCgtGGACGCTGGAAAAACAG
zju271	K435V Rev	CGACTACCAGGCATTGGAAG
zju272	T440N For	GGAAAAAacGTCTTCATGCAATCCC
zju273	T440N Rev	AGCGTCCTTGGCTCCGACTAC
zju274	E332K For	AGTaAACTTCAGAATCTCTTCTC
zju275	E332K Rev	CGGAGAAAGACATCCATC
zju148	Pcol-19-GFP-miro-1(genome) for	CAGGCTCCGAATTCGCCCTTATGAGCGACGA CGAGACGTTGG
zju149	Pcol-19-GFP-miro-1(genome) for	GCTGGGTCGAATTCGCCCTTTTACAGATTTTT CAAGACTAGG
zju238	Phsp-16.2 for	CCTGCAGGCATGCCTCGAGGGATCCAGTGAG ATGATTATAG
zju239	Phsp-16.2 rev	TAGGGCGAATTGGGTACCGTCGACTCTAGAGG

		ATCAAGAGC
zju158	Peft-3-Cas9+FZO1 sgRNA #159 for	ACTAGTCATTTTCATGGCGTGTTTTAGAGCTAGAA ATAGCAAGT
zju159	Peft-3-Cas9+FZO1 sgRNA #160 for	AGTCATTTTCATGGCGTTGGGTTTTAGAGCTAGA AATAGCAAGT
zju1047	Psur-5-dest for	ATAGGGCGAATTGGGTACCAAAGAGCGCTGTTC TTCAATTGG
zju1048	Psur-5-dest rev	CCTGCAGGCATGCCTCGAGTCTGAAAACAAAAT GTAAAG
zju3018	Pcyp-13a8-GFP for	GTAATACGACTCACTATAGGGGGAGTTTCAATT GTGCACTGG
zju3019	Pcyp-13a8-GFP rev	GACCTGCAGGCATGCCTCGAGTTTTCAGACGTT GAAAATGC
zju3020	Pcol-19-CYP-13A8 for	GCAGGCTCCGAATTCGCCCTTATGATTTTCGAG CTCATCC
zju3021	Pcol-19-CYP-13A8 rev	AGAAAGCTGGGTCGAATTCGCCCTTTCAAACTC TCGGCTTCAATGAC
zju3022	Pcyp-13a8-CYP-13A8 for	CGCGTAATACGACTCACTATAGGGCGAATTG GGTACCAATGGAAAATACCGCTACAG
zju3023	Pcyp-13a8-CYP-13A8 rev	CGACCTGCAGGCATGCCTCGAGAACTCTCGGCTTCAAT GAC
zju2612	mKate2-MIRO-1 KI test	AGGAGTCTACTACGTCGACC
**zju#**	***miro-1* sgRNA**	**sequences**
zju150	PU6-sgRNA-*miro-1* on EF-Hand I Domain for	ATTTGCGATCGAGACAACGAGT
zju151	PU6-sgRNA-*miro-1* on EF-Hand I Domain rev	TCGTTGTCTCGATCGCAAATCA
zju152	PU6-sgRNA-*miro-1* on Miro Domain for	GGGATCCGGCGAGACGTCGGGT
zju153	PU6-sgRNA-*miro-1* on Miro Domain rev	CCGACGTCTCGCCGGATCCCCA
zju154	PU6-sgRNA-*miro-1* on Rho Domain for	CTGATCGGCGACGAAGGATGGT
zju155	PU6-sgRNA-*miro-1* on Rho Domain rev	CATCCTTCGTCGCCGATCAGCA
zju156	PU6-sgRNA-*miro-1* on Trans-Membrane Domain for	GAAACCAGCTAGAGCTACTAGT
zju157	PU6-sgRNA-*miro-1* on Trans-Membrane Domain rev	TAGTAGCTCTAGCTGGTTTCCA
zju440	PU6-sgRNA-*miro-1* on EF-Hand II Domain for	AAGTACGATGAAGACAAAGAGT

zju441	PU6-sgRNA-*miro-1* on EF-Hand II Domain rev	TCTTTGTCTTCATCGTACTTCA
zju2375	miro-1 zju4 sgRNA For	TAAGATTCTCCCCATAATGGGT
zju2376	miro-1 zju4 sgRNA Rev	CCATTATGGGGAGAATCTTAAC
zju2377	miro-1 zju2 sgRNA For	CCAGGCGAATGTCATTTGTGGT
zju2378	miro-1 zju4 sgRNA Rev	CACAAATGACATTCGCCTGGAC

Fu et al., Supplementary Table 4

Outer mitochondrial membrane localization genes list for RNAi screen for WIMF regulators.

Gene name	Mitochondrial morphology before wounding	Mitochondrial morphology after wounding
abhd-11.1	small, fragmented	fragmented
acl-6	normal	fragmented
acs-1	longer	fragmented
acs-1	normal	fragmented
acs-10	normal	fragmented
acs-11	normal	fragmented
acs-12	normal	fragmented
acs-13	normal	fragmented
acs-14	normal	fragmented
acs-15	normal	fragmented
acs-16	normal	fragmented
acs-17	normal	fragmented
acs-18	normal	fragmented
acs-19	long and thin	fragmented
acs-2	normal	fragmented
acs-20	normal	fragmented
acs-21	normal	fragmented
acs-22	normal	fragmented
acs-4	long and thin	fragmented
acs-5	long and thin	fragmented
acs-6	normal	fragmented
acs-7	normal	fragmented
acs-9	normal	fragmented
akap-1	normal	fragmented
alh-12	longer	fragmented
ant-1.1	fragmented, like fzo-1 mutant	fragmented
ant-1.2	normal	fragmented
ant-1.3	normal	fragmented
ant-1.4	normal	fragmented
ant-11	thin and long, puntated	fragmented
ant-13	normal	fragmented
arx-2	normal	fragmented
atp-2	fused, large	fragmented
atp-3	fused, large	fragmented
atp-4	fused, large	fragmented
atp-5	fused, large	fragmented
bli-3	fragmented	fragmented
C16A3.10a	longer	fragmented
C30H6.7	normal	fragmented

C33A12.1	longer	fragmented
C34B2.8	fragmented, aggregated	fragmented
ced-9	normal	fragmented
cep-1	normal	fragmented
chch-3	fragmented, large	fragmented
coq-5	normal	fragmented
cpt-2	normal	fragmented
cpt-3	normal	fragmented
cpt-5	normal	fragmented
cpt-6	normal	fragmented
crls-1	elongated	fragmented
cyk-1	normal	fragmented
cyn-1	normal	fragmented
cyn-11	normal	fragmented
cyn-15	normal	fragmented
cyn-3	normal	fragmented
cyn-4	normal	fragmented
cyn-6	normal	fragmented
cyn-9	normal	fragmented
cyp-35A1	normal	fragmented
cyp-44A1	posterior fragmented and few swollen, anterior normal,	fragmented
cytb-5.1	elongated?	fragmented
cytb-5.2	longer, connect	fragmented
D2023.6	normal	fragmented
daam-1	normal	fragmented
dct-1	normal	fragmented
djr-1	thin	fragmented
drp-1	normal	fragmented
E04A4.5	normal	fragmented
eat-3	normal	fragmented
egl-1	normal	fragmented
erp-1	normal	fragmented
exc-6	normal	fragmented
F01G4.6	normal	fragmented
f17E5.2	normal	fragmented
F22B8.7	normal	fragmented
F25B5.6a	normal	fragmented
F40A3.3	normal	fragmented
F53E10.1	normal	fragmented
f55a11.4	normal	fragmented
F56A11.5	normal	fragmented
fhod-1	normal	fragmented
fhod-2	normal	fragmented

fis-1	smaller mitochondria, seems thinner and longer	fragmented
fis-1	normal	fragmented
fis-2	thinner and longer, similar to fis-1 RNAi	fragmented
fis-2	normal	fragmented
fozi-1	normal	fragmented
frl-1	normal	fragmented
fzo-1	normal	fragmented
gop-3	normal	fragmented
gpd-4	longer	fragmented
gpx-1	normal	fragmented
gsp-2	normal	fragmented
gst-42	normal	fragmented
gtl-2	normal	fragmented
his-12	elongated, arragement	fragmented
hpo-19	normal	fragmented
hxk-1	normal	fragmented
hxk-2	normal	fragmented
hxk-3	normal	fragmented
immt-1	Fragmented, round	fragmented
immt-2	Normal, longer	fragmented
irs-1	normal	fragmented
isp-1	thin	fragmented
K02F3.2	normal	fragmented
klc-1	normal	fragmented
letm-1	Fragmented, round	fragmented
lrk-1	normal	fragmented
marc-2	normal	fragmented
marc-3	small, fragmented	fragmented
marc-4	normal	fragmented
marc-5	normal	fragmented
marc-6	normal	fragmented
mcu-1	normal	fragmented
mff-1	normal	fragmented
mff-2	normal	fragmented
mics-1	normal	fragmented
micu-1	normal	fragmented
milton-1	normal	fragmented
miro-1	long and parallel	local fragmented
miro-2	long and parallel	local fragmented
miro-3	long and parallel	local fragmented
mml-1	normal	fragmented
moma-1	Fragmented, round	fragmented

mrck-1	thinner, smaller, longer mito	fragmented
mtx-1	small, fragmented	fragmented
mtx-2	elongated at L4, D1	fragmented
ncx-6	normal	fragmented
nmy-1	normal	fragmented
nmy-2	normal	fragmented
pgam-5	normal	fragmented
pgp-3	normal	fragmented
pifk-1	normal	fragmented
pink-1	normal	fragmented
R10H10.6	normal	fragmented
R11F4.1	fragmented	fragmented
rmd-1	normal	fragmented
rmd-2	normal	fragmented
rmd-3	normal	fragmented
rmd-4	normal	fragmented
rmd-5	normal	fragmented
rmd-6	normal	fragmented
rnf-5	fragmented	fragmented
rsks-1	normal	fragmented
sdha-2	longer, connected	fragmented
sec-20	normal	fragmented
sfxn-1.4	sterile, lethal, no brood size	fragmented
snb-1	normal	fragmented
snb-2	starved, small fragmented	fragmented
snb-5	normal	fragmented
snb-6	elongated	fragmented
T06D8.7	fragmented	fragmented
T10F2.2	longer	fragmented
tmem-135	normal	fragmented
Tomm-20	normal	fragmented
Tomm-22	normal	fragmented
Tomm-40	normal	fragmented
Tomm-7	normal	fragmented
unc-116	thinner mito, not similar to miro-1	fragmented
unc-16	normal	fragmented
unc-26	normal	fragmented
vdac-1	normal	fragmented
W10C8.5	normal	fragmented
wht-1	fragmented	fragmented
wsp-1	normal	fragmented
wve-1	normal	fragmented
Y40B1B.8	normal	fragmented

Fu et al., Supplementary Table 5

New *miro-1* mutations are generated using the CRISPR-Cas9 system in this study.

Allele	Mutation	sgRNA oligos
zju2	G337GGCGA deletion	ZJU2377+ZJU2378
zju3	G565A-568A substituted by CCTCCTC	ZJU2375+ZJU2376
zju4	G567AAT deletion	ZJU2375+ZJU2376
zju16	G568ATG deletion	ZJU2375+ZJU2376
zju17	G568A insertion with A	ZJU2375+ZJU2376
zju18	G569T deletion	ZJU2375+ZJU2376
zju19	G568ATGGA deletion	ZJU2375+ZJU2376
zju22	G566TAATG deletion	ZJU2375+ZJU2376
zju23	G568A deletion	ZJU2375+ZJU2376
zju24	G571G insertion with ATGG	ZJU2375+ZJU2376
zju25	G567A insertion with TCTCCCTCCC	ZJU2375+ZJU2376
zju26	G568AT substituted by GATTCTCCCCAA	ZJU2375+ZJU2376
zju27	G562CCCATAA substituted by TC	ZJU2375+ZJU2376
zju28	G567AATG deletion	ZJU2375+ZJU2376
zju29	G1620T-2534A substituted by CGGGATTTTAATCATT	ZJU152+ZJU153
zju30	G2286TCG deletion	ZJU152+ZJU153
zju33	G2278G-2293A substituted by TCGTCGGATCCCAGCGTCGT	ZJU152+ZJU153
zju34	G2281AGACGTCGGCG deletion	ZJU152+ZJU153
zju35	G1422A-2289G deletion	ZJU152+ZJU153
zju36	G2283ACGTCGGCGG deletion	ZJU152+ZJU153
zju37	G2289GCGGACG deletion	ZJU152+ZJU153
zju40	G565ATAATGG deletion	ZJU2375+ZJU2376
zju41	G563-8 substituted by TTTCCTTTCCTTTCCTT TCCTTTGATTCCTTCCTTTCCTTTGGATTCCTT	ZJU2375+ZJU2376
zju42	G564CATA deletion	ZJU2375+ZJU2376
zju43	G549T-577A substituted by GAATAAT	ZJU2375+ZJU2376
zju32	G566TAATGGA deletion	ZJU2375+ZJU2376
zju44	G55GGATGC deletion	ZJU154+ZJU155
zju45	G51CGAAGGA deletion	ZJU154+ZJU155
zju46	G57GATGCGG deletion	ZJU154+ZJU155
zju72	G762CAACGATGGA deletion	ZJU150+ZJU151
zju73	G764A-769G substituted by T	ZJU150+ZJU151
zju74	G764A-771A substituted by TGG	ZJU150+ZJU151
zju75	G763AAC deletion	ZJU150+ZJU151
zju76	G762CAACGA deletion	ZJU150+ZJU151
zju77	G764ACGATGGATATCTCA deletion	ZJU150+ZJU151

zju78	G757C-764A substituted by G	ZJU150+ZJU151
zju80	G2747-8 substituted by CTGGTAGCATAGCATA GCATAGCTGGTGCCATAGCATAGTAGGT	ZJU156+ZJU157
zju81	G2745A-2748A substituted by GGCGCT	ZJU156+ZJU157
zju82	G2747T insertion with T	ZJU156+ZJU157
zju83	G2747T-2750C substituted by CTGGTT	ZJU156+ZJU157
zju84	G2746GTA deletion	ZJU156+ZJU157
zju85	G2726TCACATTCGGCGCTGCCATAGT deletion	ZJU156+ZJU157
zju86	G2736CGCTGCCATAGTAGCTCTAGCTGG deletion	ZJU156+ZJU157
zju87	G2745AGTAGCTCTAGCTGGTTTCCTAGTC TTGAAAAATCTGTAAtagaat deletion	ZJU156+ZJU157
zju93	G2741CCATAGTAGCTCTAGCTGGTTTCCTAG deletion	ZJU156+ZJU157
zju94	G2746G-2803T substituted by ATCACATTCGG CGCTGCCATCACATTCGGCGCTGCCATA	ZJU156+ZJU157
zju95	G2737GCTGCCATAGTAGCTC deletion	ZJU156+ZJU157
zju138	G1123AAA substituted by G	ZJU440+ZJU441
zju139	G814T-1125A substituted by GATG	ZJU440+ZJU441
zju140	G1125A deletion	ZJU440+ZJU441
zju141	G1125A substituted by TGATAAGACAATGAT	ZJU440+ZJU441
zju142	G1123A insertion with G	ZJU440+ZJU441
zju143	G1122CAAAGA deletion	ZJU440+ZJU441
zju144	G1123AAAGAT deletion	ZJU440+ZJU441
zju148	G1124A insertion with TCGTCTTCATGATGA	ZJU440+ZJU441
zju149	G1122CAAAGATGGATGT deletion	ZJU440+ZJU441
zju150	G1124AA deletion	ZJU440+ZJU441
zju151	G1108AAGTACGATGAAGACAAAGA deletion	ZJU440+ZJU441
zju157	G1124AA deletion	ZJU440+ZJU441
zju158	G1124AAGA deletion	ZJU440+ZJU441
zju159	G1125A deletion	ZJU440+ZJU441
zju160	G1125A insertion with AGTACGATGAAGAC	ZJU440+ZJU441
zju161	G1123AAA deletion	ZJU440+ZJU441
zju163	G1123A-1128T substituted by GATGATGA	ZJU440+ZJU441
zju164	G1122 CAAAGA deletion	ZJU440+ZJU441
zju173	G1101A-1128T deletion	ZJU440+ZJU441

Fu et al., Supplementary Table 6

Comparison of MiST and WIMF

**Content**	**MiST**	**WIMF**	**Key data in Nemani et al., paper [10]**	**Key data in our paper**
**Model**	Cells	Cells and Animals	Fig. 1	Fig.1, Fig. S1
**Time**	Minutes	Seconds	Fig. 1B, F,	Fig. 1, Fig. S1
**Fission machinery**	Independent on DRP-1	Inhibited by DRP-1	Fig. 4B, 4D-F	Fig. S3b
**MIRO-1 GTPase**	dispensable	required	Fig. S5I–S5N	Fig. 4b
**Outcome**	Autophagy and mitophagy	ROS signaling, Gene expression,	Fig. 7A-D	Fig. 7
**Physiological significant**	mitochondrial removal	Promotes wound repair	Fig. 7G-H	Fig. 2, Fig. 5, Fig. 7
**Recovery**	No	Yes	Fig. 7	Fig. 1d

Supplementary Table 6: Comparison of MiST and WIMF. Nemani et al., find that mitochondria undergo mitochondrial shape transition (MiST) upon Ca^{2+} stress and GPCR activation [10]. They showed that MiST is distinct from MCU-induced swelling and mitochondrial dynamics. The conserved Ca^{2+} sensor Miro1 EF-1 enables MiST and promotes autophagy/mitophagy. Thus, MiST and WIMF (Wounding-Induced Mitochondrial Fragmentation) share some similarities. For example, both processes induce mitochondrial fragmentation/shape change, both require Ca^{2+}-MIRO-1 signaling, and both are independent of mitochondrial Ca^{2+} uptake. However, after carefully comparing both processes, we consider that WIMF is different from MiST. For example, in time courses, MiST happens in minutes after Ca^{2+} treatment, whereas WIMF happens in seconds after wounding. Although MIRO-1 is required for both processes, the MIRO-1 GTPase domain is dispensable for MiST but is required for WIMF. MiST is distinct from mitochondrial fission and swelling; however, WIMF not only including mitochondrial shape change but also included mitochondrial fission and swelling. The outcomes of the two processes are also significantly different: MiST induces autophagy or mitophagy to remove the damaged mitochondria, while WIMF triggers ROS production, affects gene expression and accelerates wound repair. Besides, mitochondrial morphology can be fully recovered after WIMF but not after MiST.

Supplementary References:

1. Rolland, S.G., Lu, Y., David, C.N. & Conradt, B. The BCL-2-like protein CED-9 of C. elegans promotes FZO-1/Mfn1,2- and EAT-3/Opa1-dependent mitochondrial fusion. *J Cell Biol* **186**, 525-540 (2009).

2. Delivani, P., Adrain, C., Taylor, R.C., Duriez, P.J. & Martin, S.J. Role for CED-9 and Egl-1 as regulators of mitochondrial fission and fusion dynamics. *Mol Cell* **21**, 761-773 (2006).

3. Breckenridge, D.G., Kang, B.H. & Xue, D. Bcl-2 proteins EGL-1 and CED-9 do not regulate mitochondrial fission or fusion in Caenorhabditis elegans. *Curr Biol* **19**, 768-773 (2009).

4. Ramabhadran, V., Hatch, A.L. & Higgs, H.N. Actin monomers activate inverted formin 2 by competing with its autoinhibitory interaction. *J Biol Chem* **288**, 26847-26855 (2013).

5. Korobova, F., Ramabhadran, V. & Higgs, H.N. An actin-dependent step in mitochondrial fission mediated by the ER-associated formin INF2. *Science* **339**, 464-467 (2013).

6. Xu, S. & Chisholm, A.D. A Galphaq-Ca(2)(+) signaling pathway promotes actin-mediated epidermal wound closure in C. elegans. *Curr Biol* **21**, 1960-1967 (2011).

7. Xu, S. & Chisholm, A.D. C. elegans epidermal wounding induces a mitochondrial ROS burst that promotes wound repair. *Dev Cell* **31**, 48-60 (2014).

8. Pujol, N. et al. Distinct innate immune responses to infection and wounding in the C. elegans epidermis. *Curr Biol* **18**, 481-489 (2008).

9. Zhou, J. et al. The lysine catabolite saccharopine impairs development by disrupting mitochondrial homeostasis. *J Cell Biol* (2018).

10. Nemani, N. et al. MIRO-1 Determines Mitochondrial Shape Transition upon GPCR Activation and Ca(2+) Stress. *Cell Rep* **23**, 1005-1019 (2018).
