# Supplementary material for: Wounding triggers MIRO-1 dependent mitochondrial fragmentation that accelerates epidermal wound closure through oxidative signaling
Source: Nat Commun. 2020 Feb 26;11:1050. doi: 10.1038/s41467-020-14885-x (PMC7044169; doi:10.1038/s41467-020-14885-x)
Supplement: Supplementary file 3 — Description of Additional Supplementary Files [file 41467_2020_14885_MOESM3_ESM.pdf]

Description of Additional Supplementary Files

File Name: Supplementary Data 1
Description: RNA-sequencing detects wounding-induced genes in wild type and fzo-1 mutant animals

File Name: Supplementary Data 2
Description: Overlapping genes in wounded wild type and fzo-1 mutant animals

File Name: Supplementary Movie 1
Description: **Mitochondrial morphology in the adult *C. elegans* epidermis before and after femtosecond laser wounding. Related Fig. 1b.**
Epidermal mitochondria are tubular and form a stable reticulum. mito::GFP was expressed in the *C. elegans* adult epidermis (*juEx4796, col-19* promoter). Images were taken on the spinning disk confocal microscope. After laser wounding, mitochondria become fragmented and swollen close to the wound site. Scale bar, 10 μm. Time: sec.

File Name: Supplementary Movie 2
Description: **Mitochondrial morphology in the adult *C. elegans* epidermis before and after Micropoint UV laser wounding. Related to Fig. 1c.**
Epidermal mitochondria are tubular and form a stable reticulum. P*col-19-mito::GFP*(*juEx4796*) was expressed in the *C. elegans* adult epidermis. Images were taken every 2 s on the spinning disk confocal microscope. After Micropoint UV laser wounding, mitochondria become fragmented and swollen close to the wound site. Scale bar, 10 μm. Time: sec.

File Name: Supplementary Movie 3
Description: **Wounding triggers actin polymerization at the wound site in the wild type animals. Related to Supplementary Fig. 2a.**
Pcol-19-GFP::moesin (juIs352) labels epidermal F-actin, *mito::mKate2(zjuSi47) l*abels epidermal mitochondria. Scale bar: 10 μm; Time: min.

File Name: Supplementary Movie 4
Description: **Wounding triggers actin-based wound closure in WT and accelerated closure in the *fzo-1* mutants. Related to Fig. 2a and Supplementary Fig. 2b.**
GFP::moesin *(juIs352)* labels epidermal F-actin. The *fzo-1(tm1133);Pcol-19-GFP::moesin(juIs352)* mutant shows faster wound closure compared to WT. Scale bar: 10 μm; Time: min.

File Name: Supplementary Movie 5
Description: **Laser wounding-induced mitochondrial fragmentation is independent of mitochondrial fission protein DRP-1. Related to Supplementary Fig. 3b.**
Mitochondria were largely connected in the *drp-1(tm1108)* mutant animals. Laser wounding caused fragmentation of mitochondria in both WT and *drp-1* mutant. The fragmented mitochondria extend farther in *drp-1* than in WT after wounding. Mitochondria were labeled by P*col-19-mito::dendra2(juSi271)*. Scale bar: 10 μm. Time: sec.

File Name: Supplementary Movie 6
Description: **Mitochondrial morphology in *miro-1(tm1966)* mutant before and after Micropoint UV laser wounding. Related to Fig. 3c.**
Pcol-19-mito::dendra2(juSi271) was expressed in the adult epidermis to label the mitochondria. Images were taken every 10 s on the spinning disk confocal microscope. Red signals were photoconverted before wounding using 405 nm laser. After Micropoint UV laser wounding, mitochondria fragmentation could spread to the neighbor region in WT but not in the *miro-1* mutant. Scale bar, 10 μm. Time: sec.

File Name: Supplementary Movie 7
Description: **The localization of GFP::MIRO-1 and GFP::MIRO-1(EF-hand mutation) before and after wounding. Related to Supplementary Fig. 5b**
Pcol-19-GFP::miro-1;Pcol-19-mito::mkate2(zjuEx57), Pcol-19-GFP::miro-1(EF-hand mutation);Pcol-19-mito::mkate2(zjuEx59), were expressed in the epidermis. The epidermis of the animal was wounded by Micropoint UV laser. Scale bar: 10 μm. Time: sec.

File Name: **Supplementary Movie 8**
Description: **Mitochondrial and microtubule dynamics in WT *C. elegans* epidermis before wounding. Related to Supplementary Fig. 4f.**
P*col-19-mito::crimson;*P*col-19-GFP::tbb-2(juIs492)* animal was imaged using a spinning disk confocal microscope. Scale bar: 10 μm. Time: sec.

File Name: **Supplementary Movie 9**
Description: **Wounding triggers mitochondrial fragmentation and microtubule depolymerization. Related to Supplementary Fig. 4g.** P*col-19-mito::crimson;*P*col-19-GFP::tbb-2(juIs492)* animal was imaged using spinning disk confocal microscope and wounded by Micropoint UV laser. Scale bar: 10 μm. Time: sec.

File Name: Supplementary Movie 10
Description: **Wounding triggers mitochondrial fragmentation and microtubule depolymerization in the *miro-1* mutant. Related to Supplementary Fig. 4g.**
P*col-19-mito::crimson;*P*col-19-GFP::tbb-2(juIs492);miro-1(tm1966)* animal was imaged using spinning disk confocal microscope and wounded by Micropoint UV laser. Scale bar: 10 μm. Time: sec.

File Name: Supplementary Movie 11
Description: **MIRO-1's Rho, EF-hand, and TM domain are required for WIMF. Related to Supplementary Fig. 5f.**
P*col-19-mito::dendra2(juSi271);miro-1(zju44) ΔRho,*
Pcol-19-mito::dendra2(juSi271); miro-1(zju84) ΔMiro,
P*col-19-mito::dendra2(juSi271);miro-1(zju75,zju162) ΔEF-hand,*
P*col-19-mito::dendra2(juSi271);miro-1(zju87) ΔTM* animals were imaged using spinning disk confocal microscope and wounded by Micropoint UV laser. Scale bar: 10 μm. Time: sec.

File Name: Supplementary Movie 12
Description: **Laser wounding triggers mitochondrial fragmentation in *gtl-2(n2618)* and *mcu-1(ju1154)* mutants. Related to Supplementary Fig. 5f.**
P*col-19-mito::dendra2(juSi271);gtl-2(n2618) and* P*col-19-mito::dendra2(juSi271);mcu-1(ju1154) mutant* animals were imaged using spinning disk confocal microscope and wounded by Micropoint UV laser. Scale bar: 10 μm. Time: sec.

File Name: Supplementary Movie 13
Description: **Laser wounding triggers mitochondrial fragmentation in *gtl-2(n2618),* and ionomycin treated animals. Related to Fig. 4e.**
P*col-19-mito::dendra2(juSi271);gtl-2(n2618)* animals were treated with 2.5 μM ionomycin for 3 hours and were imaged using spinning disk confocal microscope and wounded by Micropoint UV laser. Scale bar: 10 μm. Time: sec.

File Name: **Supplementary Movie 14**
Description: **Loss of FZO-1 reduced wounding induced activation of RHO-1 at the wound site. Related to Fig. 7f.**
Laser wounding of P*col-19*-eGFP::rGBD*(juEx3025)* worms in WT, *drp-1(tm1108)* and *fzo-1(tm1133)* mutants, intensity color code. Scale bar: 10 μm. Time: sec.
