# Supplementary material for: Wounding triggers MIRO-1 dependent mitochondrial fragmentation that accelerates epidermal wound closure through oxidative signaling
Source: Nat Commun. 2020 Feb 26;11:1050. doi: 10.1038/s41467-020-14885-x (PMC7044169; doi:10.1038/s41467-020-14885-x)
Supplement: Supplementary file 20 — Reporting Summary [file 41467_2020_14885_MOESM20_ESM.pdf]

nature research

Corresponding author(s): Suhong Xu

Last updated by author(s): Jan 27, 2020

Reporting Summary

Nature Research wishes to improve the reproducibility of the work that we publish. This form provides structure for consistency and transparency in reporting. For further information on Nature Research policies, see Authors & Referees and the Editorial Policy Checklist.

Statistics

For all statistical analyses, confirm that the following items are present in the figure legend, table legend, main text, or Methods section.

n/a	Confirmed	
☐	☒	The exact sample size (*n*) for each experimental group/condition, given as a discrete number and unit of measurement
☐	☒	A statement on whether measurements were taken from distinct samples or whether the same sample was measured repeatedly
☐	☒	The statistical test(s) used AND whether they are one- or two-sided<br>*Only common tests should be described solely by name; describe more complex techniques in the Methods section.*
☐	☒	A description of all covariates tested
☒	☐	A description of any assumptions or corrections, such as tests of normality and adjustment for multiple comparisons
☐	☒	A full description of the statistical parameters including central tendency (e.g. means) or other basic estimates (e.g. regression coefficient) AND variation (e.g. standard deviation) or associated estimates of uncertainty (e.g. confidence intervals)
☐	☒	For null hypothesis testing, the test statistic (e.g. *F*, *t*, *r*) with confidence intervals, effect sizes, degrees of freedom and *P* value noted<br>*Give P values as exact values whenever suitable.*
☒	☐	For Bayesian analysis, information on the choice of priors and Markov chain Monte Carlo settings
☒	☐	For hierarchical and complex designs, identification of the appropriate level for tests and full reporting of outcomes
☒	☐	Estimates of effect sizes (e.g. Cohen's *d*, Pearson's *r*), indicating how they were calculated

Our web collection on statistics for biologists contains articles on many of the points above.

Software and code

Policy information about availability of computer code

Data collection	Andor Spinning Disk confocal microscope and IQ image software (IQ CORE)
Data analysis	Image analysis: MetaMorph (7.8) and image J software (1.44).<br>Statistic analysis: Graphpad prism (7.0)<br>RNAseq analysis:<br>1. STAR<br>2. R subreads package<br>3. DESeq2<br>4. DAVID<br>5. ggplot2 package in R<br>6. Complex heatmap package in R

For manuscripts utilizing custom algorithms or software that are central to the research but not yet described in published literature, software must be made available to editors/reviewers. We strongly encourage code deposition in a community repository (e.g. GitHub). See the Nature Research guidelines for submitting code & software for further information.

Data

Policy information about availability of data

All manuscripts must include a data availability statement. This statement should provide the following information, where applicable:

- Accession codes, unique identifiers, or web links for publicly available datasets
- A list of figures that have associated raw data
- A description of any restrictions on data availability

The authors declare that all data supporting the findings of this study are available within this article, its supplementary Information files, the peer-review file, the source data file, or are available from the corresponding author upon reasonable request.

The RNA sequencing data generated and analyzed in this study are available upon request as well as from the Sequence Read Archive (SRA) at NCBI at the following accession code: PRJNA523321(https://www.ncbi.nlm.nih.gov/sra/?term=PRJNA5 23321)

mitoCarta database is available here: https://www.broadinstitute.org/files/shared/metabolism/mitocarta/human. mitocarta2.0.html

Field-specific reporting

Please select the one below that is the best fit for your research. If you are not sure, read the appropriate sections before making your selection.

☒ Life sciences ☐ Behavioural & social sciences ☐ Ecological, evolutionary & environmental sciences

For a reference copy of the document with all sections, see nature.com/documents/nr-reporting-summary-flat.pdf

Life sciences study design

All studies must disclose on these points even when the disclosure is negative.

Sample size	Sample size (Fig.1e,2b,2d,2g,2j, 3d,4b,4c,4e,5a,5b,6n-p,7b,7d,7e,7g, Fig. S1d, S2c-f,S3c,S4f,S4g, S5a,S5h,S7a, S7d-e, S7g, S7h) were determined as numbers of animals that have been successfully experimented and imaged. The sample size of qPCR (Fig. 6e-l, 7c, S6e) were determined as number of independent experiments. Sample size of Fig. S1f were determined by the number of cell analyzed.
Data exclusions	No data were excluded from the analysis.
Replication	Attempts at least 2 replication were performed successfully.
Randomization	For all experiments, animals were randomly allocated to the experimental groups
Blinding	The investigators were not blinded to allocation during experiments and outcome assessment. Some data analysis (Fig.1e, Fig. 2b-d, Fig. 3d, Fig.7e) was blinded to observer. For most of the experiment, at least one negative control group was set to ensure that only one variate was tested. And because the data were conducted based on random sampling and reasonablly large sample size, blinding is not relevant to this study.

Reporting for specific materials, systems and methods

We require information from authors about some types of materials, experimental systems and methods used in many studies. Here, indicate whether each material, system or method listed is relevant to your study. If you are not sure if a list item applies to your research, read the appropriate section before selecting a response.

Materials & experimental systems

n/a	Involved in the study	
☒	☐	Antibodies
☐	☒	Eukaryotic cell lines
☒	☐	Palaeontology
☐	☒	Animals and other organisms
☒	☐	Human research participants
☒	☐	Clinical data

Methods

n/a	Involved in the study	
☒	☐	ChIP-seq
☒	☐	Flow cytometry
☒	☐	MRI-based neuroimaging

Eukaryotic cell lines

Policy information about cell lines

Cell line source(s)	Commercial cell lines U2OS from ATCC
Authentication	U2OS cell line was bought from ATCC but were not authenticated.
Mycoplasma contamination	U2OS cell line was tested negative for mycoplasma contamination
Commonly misidentified lines (See ICLAC register)	No cell lines used in this study were found in the database of commonly misidentified cell lines that is maintained by ICLAC and NCBI Biosample.

Animals and other organisms

Policy information about studies involving animals; ARRIVE guidelines recommended for reporting animal research

Laboratory animals	C. elegans (All the strains used are described in Supplementary Table 1, animals were adult hermaphrodites), Zebrafish (AB line, 3 day larvae were used)
Wild animals	None of the wild animals used
Field-collected samples	None
Ethics oversight	In China, no ethics approval is needed for experiments using C. elegans or Zebrafish larvae.

Note that full information on the approval of the study protocol must also be provided in the manuscript.
